# Dynamic interplay between catalytic and lectin domains of GalNAc-transferases modulates protein *O*-glycosylation

**DOI:** 10.1038/ncomms7937

**Published:** 2015-05-05

**Authors:** Erandi Lira-Navarrete, Matilde de las Rivas, Ismael Compañón, María Carmen Pallarés, Yun Kong, Javier Iglesias-Fernández, Gonçalo J. L. Bernardes, Jesús M. Peregrina, Carme Rovira, Pau Bernadó, Pierpaolo Bruscolini, Henrik Clausen, Anabel Lostao, Francisco Corzana, Ramon Hurtado-Guerrero

**Affiliations:** 1 BIFI, University of Zaragoza, BIFI-IQFR (CSIC) Joint Unit, Mariano Esquillor s/n, Campus Rio Ebro, Edificio I+D, Zaragoza 50018, Spain; 2 Departamento de Química, Universidad de La Rioja, Centro de Investigación en Síntesis Química, E-26006 Logroño, Spain; 3 LMA, INA, Universidad de Zaragoza, 50018 Zaragoza, Spain; 4 Copenhagen Center for Glycomics, Department of Cellular and Molecular Medicine, School of Dentistry, University of Copenhagen, Copenhagen DK-2200, Denmark; 5 Departament de Química Orgànica i IQTCUB, Universitat de Barcelona, Martí i Franquès 1, 08028 Barcelona, Spain; 6 Department of Chemistry, University of Cambridge, Lensfield Road, Cambridge CB2 1EW, UK; 7 Instituto de Medicina Molecular, Faculdade de Medicina, Universidade de Lisboa, Av Prof Egas Moniz, 1649-028 Lisboa, Portugal; 8 ICREA, Passeig Lluís Companys 23, 08020 Barcelona, Spain; 9 Centre de Biochimie Structurale, INSERM U1054, CNRS UMR 5048, Université Montpellier 1 and 2, 29 rue de Navacelles, 34090 Montpellier, France; 10 Departamento de Física Teórica, Universidad de Zaragoza, Zaragoza 50009, Spain; 11 Fundación ARAID, 50018 Zaragoza, Spain

## Abstract

Protein *O*-glycosylation is controlled by polypeptide GalNAc-transferases (GalNAc-Ts) that uniquely feature both a catalytic and lectin domain. The underlying molecular basis of how the lectin domains of GalNAc-Ts contribute to glycopeptide specificity and catalysis remains unclear. Here we present the first crystal structures of complexes of GalNAc-T2 with glycopeptides that together with enhanced sampling molecular dynamics simulations demonstrate a cooperative mechanism by which the lectin domain enables free acceptor sites binding of glycopeptides into the catalytic domain. Atomic force microscopy and small-angle X-ray scattering experiments further reveal a dynamic conformational landscape of GalNAc-T2 and a prominent role of compact structures that are both required for efficient catalysis. Our model indicates that the activity profile of GalNAc-T2 is dictated by conformational heterogeneity and relies on a flexible linker located between the catalytic and the lectin domains. Our results also shed light on how GalNAc-Ts generate dense decoration of proteins with *O*-glycans.

## Abstract (web summary)

Polypeptide GalNAc-transferases decorate proteins with dense arrays of *O*-glycans, which in the case of mucins are essential for their barrier functions. Here the authors present comprehensive structural studies that shed light on the molecular attributes that allow GalNAc-T2 to efficiently carry out dense *O*-glycosylation.

## Graphical abstract

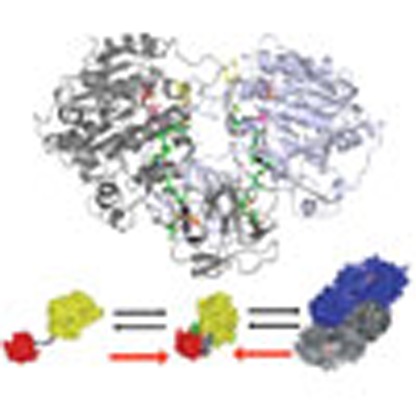

Mucin-type (GalNAc-type) *O*-glycosylation is by far the most differentially and complex regulated type of protein glycosylation, and likely the most abundant with over 80% of all proteins passing through the secretory pathway that is predicted to be *O*-glycosylated^1^. In metazoans, this post-translational modification is initiated by a large family (20 in humans) of polypeptide *N*-acetylgalactosaminyltransferases (GalNAc-Ts), which transfer a GalNAc residue from uridine diphosphate *N*-acetylgalactosamine (UDP-GalNAc) to Ser/Thr side chains in the presence of manganese^2^. These isoenzymes are grouped in CAZy database as family 27 (ref. 3) and further classified into several subfamilies based on the enzyme protein sequences and the genomic structures of the encoding genes^2^. They are also classified into two major classes based on their primary acceptor substrate preferences for peptides and partially glycosylated GalNAc-glycopeptides. The acceptor substrate specificities of these isoenzymes are distinct but partly overlapping, and so far no clear global consensus motifs or isoform-specific motifs that govern protein *O*-glycosylation have emerged, although improved algorithms for predictions have been proposed^1^,^4^. These isoenzymes have different cell and tissue expression patterns^2^ and they play important roles in health and disease^5^,^6^. The immediate product of the GalNAc-Ts is also known as the Tn (GalNAcα1-*O*-Ser/Thr) antigen (if sialylated the STn (NeuAcα2-3GalNAcα1-*O*-Ser/Thr) antigen), and while this structure in normal cells is masked by elongation of the glycan structures by a number of other glycosyltransferases, expression of Tn (and STn) is a hallmark of cancer cells^7^,^8^. Expression of these immature truncated *O*-glycans is strongly correlated with poor prognosis and low overall survival and truncated *O*-glycans serve as targets for immunotherapies^9^. Mechanisms leading to expression of Tn and STn *O*-glycans in cancers include somatic mutations and/or epigenetic silencing of the private chaperone Cosmc responsible for *O*-glycan elongation^10^ as well as relocation of GalNAc-Ts from Golgi to endoplasmic reticulum^11^. More recently, we have shown that the truncated *O*-glycophenotype appear to directly induce oncogenic features with enhanced growth and invasion^12^.

GalNAc-T isoforms contain an *N*-terminal catalytic domain adopting a GT-A fold and a unique C-terminal lectin domain, classified as a carbohydrate-binding module (CBM) 13 in the CAZy database^3^, with a β-trefoil fold that are connected through a short flexible linker^13^,^14^,^15^,^16^. A key structural feature of these enzymes in catalysis is the existence of a flexible loop formed by residues Arg362 to Ser373 (residue numbers in GalNcAc-T2) that adopts different conformations during the catalytic cycle and renders the enzyme catalytically inactive or active^16^.

Another level of complexity in this family of enzymes comes from the lectin domains that play a very important role in the *O*-glycosylation process by modulating the specificity and the use of partially glycosylated GalNAc-glycopeptide substrates^17^,^18^. Lectin domains or CBMs also exist in some glycosyl hydrolases (GHs). For these enzymes, CBMs are important for targeting the catalytic GHs onto their substrates and enhancing their hydrolytic activity, and even can potentiate the action of a cognate catalytic module towards polysaccharides in intact cell walls through the recognition of nonsubstrate polysaccharides^19^,^20^. We have hypothesized that the lectin domains of GalNAc-Ts are required to accommodate efficient glycosylation of diverse protein sequences with densely located acceptor sites in a process where the lectin domains bind to initially attached GalNAc residues and promote catalytic efficiency and further incorporation to less efficient acceptor sites^2^,^21^. The importance of the lectin domains is exemplified by the rare disease familial tumoral calcinosis associated with hyperphosphatemia, which is caused by deficiency in GalNAc-T3 or FGF23 (ref. 22). Thus, loss of GalNAc-T3, which mediates the *O*-glycosylation of Thr178 in a proprotein convertase-processing site (RHTR^179^↓) of FGF23, results in inactivation of FGF23 and cause hyperphosphatemia^22^. Notably, glycosylation of Thr178 in FGF23 by GalNAc-T3 requires the lectin domain and a GalNAc *O*-glycan positioned *N*-terminal to this site^5^. However, the mechanism for how these unique lectins modulate *O*-glycosylation is essentially unknown. In this regard, recent studies have provided evidence that the *N*/*C*-terminal orientation of GalNAc residues and available proximal acceptor sites is an important factor in the overall catalytic activity and specificity of GalNAc-Ts, and moreover, that the preferences for this orientation differs between isoforms^23^. This adds another level of differential regulation and complexity to the *O*-glycosylation process and help to explain how large mucins with thousands of glycosites are densely decorated with *O*-glycans with high fidelity. Interestingly, studies with random glycopeptide libraries suggest that there is a distance preference for the effect of prior GalNAc residues, in which the optimal distance from the prior site of glycosylation corresponds to residues that are located approximately ten residues apart for GalNAc-T1, T2 and T13, and eight or nine residues for T3 (ref. 23). This distance preference suggests a cooperative binding of GalNAc-Ts to GalNAc-glycopeptide substrates through both the catalytic and lectin domains^23^.

Here we present the first crystal structures of the inactivated and activated forms of the GalNAc-T2 isoform in binary and ternary complexes with different GalNAc-glycopeptides enabling us to study in detail the lectin-mediated modulatory mechanism of catalysis. The crystal structures, in combination with metadynamics simulations, provides a rational explanation of why this isoform favours glycosylation of acceptor sites positioned at the *N*-terminal region and ten residues apart from the prior GalNAc moieties. We also present single-molecule atomic force microscopy (AFM) and small-angle X-ray scattering (SAXS) data indicating that GalNAc-Ts sample a highly complex conformational landscape formed by ensembles of compact and extended structures. Analysis of SAXS shows that alterations of the equilibrium towards the compact structures in the presence of acceptor substrates appear to be required for lectin-mediated catalysis. In addition, by using a coarse-grained model, we demonstrate that GalNAc-T2 structural heterogeneity, as well as its activity profile, can be simply related to the mechanical properties of the flexible linker.

## Results

### Architecture of the GalNAc-glycopeptides complexes

**Figure 1:**
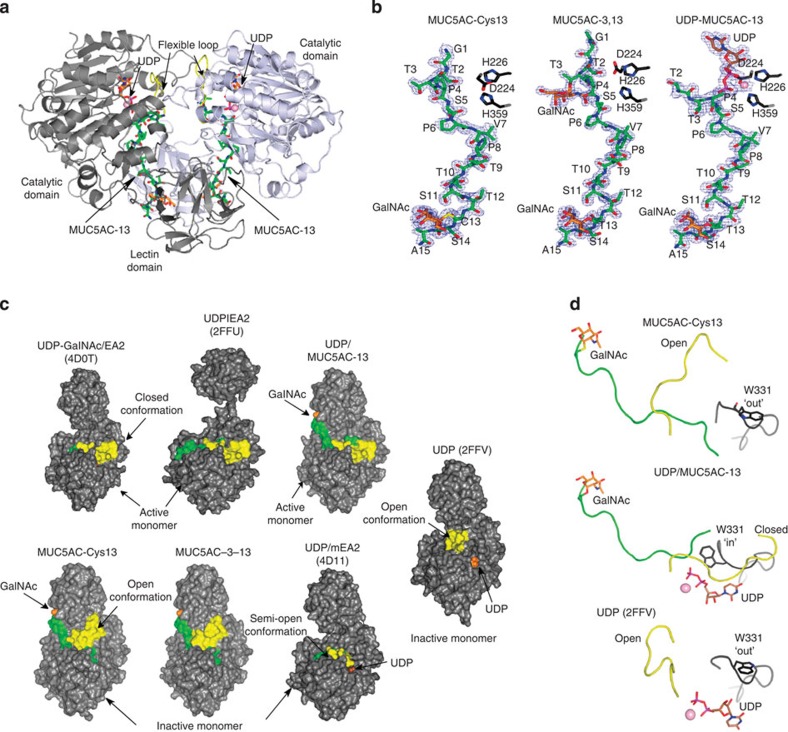
Crystal structures of GalNAc-T2 in complex with glycopeptides. (**a**) Cartoon representation of the overall dimeric structure of GalNAc-T2 in complex with UDP-MUC5AC-13. The monomers are coloured in grey and blue-white, respectively. UDP, MUC5AC-13 and flexible loop in yellow are indicated by arrows. The nucleotide and the glycopeptide are depicted as orange and green carbon atoms, respectively. The GalNAc moiety covalently bound to Thr13 is depicted as orange carbon atoms. (**b**) Electron density maps are F_O_–F_C_ syntheses (blue) contoured at 2.0*σ* for the ligands. The residues (Asp224, His226 and His359) coordinated to the manganese atom (shown as a pink sphere) are illustrated as black carbon atoms. Colours for the ligands are the same as above. (**c**) Surface representation of binary and ternary GalNAc-T2 complexes showing different active and inactive states. Protein, flexible loop, nucleotides, GalNAc moiety and peptides are coloured in grey, yellow, brown, orange and green, respectively. The active and inactive states are indicated as closed and open conformations, respectively. (**d**) Close-up view of GalNAc-T2-MUC5AC-Cys13, GalNAc-T2-MUC5AC-13-UDP and GalNAc-T2-UDP (PDB entry 2FFV^14^) complexes. The colours are the same as shown above. The loop in which Trp331 is located is in black. In the latter complex, UDP is shown as inverted relative to the most frequent position found for UDP and has been proposed to be a conformation ready to depart from the active site^14^,^16^. Trp331 is depicted as black carbon atoms and adopts ‘in' and ‘out' conformations.

To understand how lectin domains direct *O*-glycosylation, we obtained tetragonal crystals of GalNAc-T2 in complex with three GalNAc-glycopeptides (Table 1 and Supplementary Table 1). The resulting crystals allowed us to solve the structure at high resolution (from 1.48 to 1.67 Å) and easily interpret the density maps (Table 1). Despite the co-crystallization experiments were performed with GalNAc-T2, UDP and Mn^+2^, we obtained binary complexes with glycopeptides MUC5AC-Cys13 and MUC5AC-3-13 (the binary complexes are referred to the enzyme complexed to the different glycopeptides MUC5AC-Cys13 and MUC5AC-3-13, respectively), and a ternary complex with MUC5AC-13 (the ternary complex is referred to the enzyme bound to UDP and the glycopeptide MUC5AC-13; Fig. 1a,b).

Within the asymmetric unit (AU) of I4_1_ crystals, one molecule of GalNAc-T2 is arranged as a dimer with another molecule from the neighbouring AU (Fig. 1a). These compact dimeric forms are consistent with previous structures of GalNAc-T2 in complexes with UDP and Mn^+2^ (protein data bank (PDB) entry 2FFV^14^), and UDP or UDP-GalNAc/Mn^+2^ and peptides (PDB entries 4D0T, 4D0Z and 4D11 (ref. 16)). However, members of this family of enzymes are proposed to be monomers in solution^16^,^24^,^25^ (discussed below). The crystal structures show that the typical GT-A fold is located in the *N*-terminal region and the lectin domain is located in the *C*-terminal region (Fig. 1a). The density for all the glycopeptides and UDP/Mn^+2^ was well defined in most monomers (Fig. 1b). These results are further supported by tryptophan fluorescence spectroscopy experiments showing that peptides/glycopeptides can bind fairly well to GalNAc-T2 in the absence or presence of UDP/Mn^+2^ (Supplementary Table 2). The dissociation constants (*K*_*d*_s) of the peptides were in the low μM range and very similar among them except for the naked peptide MUC5AC, in which the *K*_*d*_ in the absence of UDP/Mn^+2^ is fourfold better than in the presence of the nucleotide (Supplementary Table 2).

The structures also feature a flexible loop that can oscillate between closed and open conformations (Fig. 1c,d) and is associated with the active and inactive states of the enzyme, as described in previous studies^14^,^16^. We also reported that the UDP-GalNAc moiety was a key factor to maintain the flexible loop in a closed conformation regardless of whether the peptide was present or not (Fig. 1c and PDB entry 4D0T)^16^. On the contrary, previous structures containing UDP (Fig. 1c and PDB entries 2FFU^14^, 4D11 (ref. 16) and 2FFV^14^) including our complex of GalNAc-T2-UDP-MUC5AC-13 (Fig. 1c,d) exhibit a mix of loop conformations (semi-open, open and closed) along the catalytic cycle (Fig. 1c)^16^. Furthermore, the flexible loop dynamics are coupled to the key catalytic residue Trp331 mobility that can adopt ‘in' (inside of the active site) and ‘out' (outside of the active site) conformations, which in turn are associated to the active or inactive states of GalNAc-T2, respectively (Fig. 1d). Interestingly, MUC5AC-13 was bound to an active form of GalNAc-T2, whereas MUC5AC-Cys13 and MUC5AC-3-13 were unpredictably trapped bound to the inactive form of GalNAc-T2 (Fig. 1b–d). In these cases, the flexible loop was found in an open conformation, different from the one previously described for the GalNAc-T2-UDP complex (PDB entry 2FFV^14^ and root-mean-square deviation (RMSD) of 4.68 Å for aligned Cα atoms corresponding to the flexible loop; Fig. 1c,d), which further demonstrates the loop versatility.

### Peptide and lectin domain-binding sites

**Figure 2:**
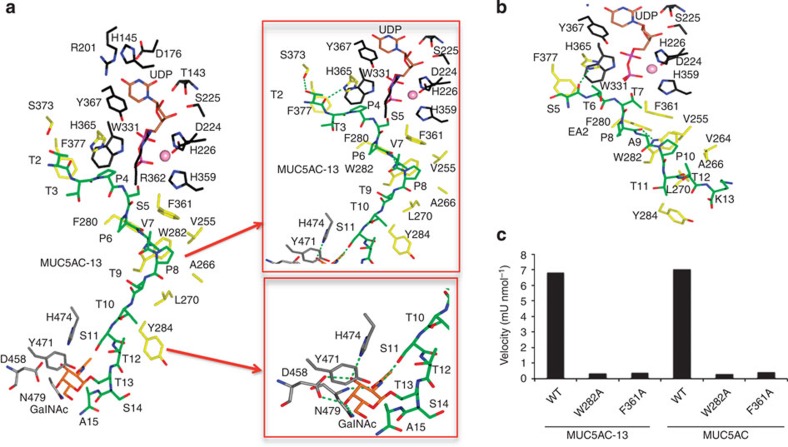
Structural features of peptide and lectin domain-binding sites. (**a**) View (left panel) of complete sugar nucleotide, peptide and lectin domain-binding sites of the GalNAc-T2-UDP-MUC5AC-13 complex. Close-up view (right panel) of peptide and lectin domain-binding sites. The residues forming sugar-nucleotide, peptide and lectin domain-binding sites are depicted as black, yellow and grey carbon atoms, respectively. UDP and the glycopeptide are shown as brown and green carbon atoms, respectively. Mn^+2^ and GalNAc moiety are depicted as a pink sphere and orange carbon atoms, respectively. Hydrogen bond interactions are shown as dotted green lines. (**b**) Close-up view of the peptide-binding site of the GalNAc-T2-UDP-EA2 complex. Colours are the same as above. (**c**) Graph that shows the velocity for the wild-type (WT) enzyme and mutants with peptides MUC5AC and MUC5AC-13. Time-course experiments were carried out and the reactions were analysed and evaluated by MALDI-TOF-MS. The specific activities under linear conditions were inferred from the mass spectrometry data (see Methods for details). One unit of enzyme is defined as the amount of enzyme that transfers 1 μmol of GalNAc in 1 min using the standard reaction mixture and conditions. The velocity values were obtained from three independent experiments and errors are <20%.

As shown in the crystal structures (Fig. 1), the GalNAc-T2-binding site is large and set up by three regions: the sugar nucleotide, the peptide and the lectin domain-binding sites (Fig. 2a). In the GalNAc-T2-MUC5AC-13-UDP complex, GalNAc-T2 is in an active state with the flexible loop in a closed conformation with UDP in the sugar nucleotide-binding site, and MUC5AC-13 acts as a bridge between the catalytic unit and the lectin domain (Fig. 2a). MUC5AC-13 as well as MUC5AC-Cys13 and MUC5AC-3-13 glycopeptides have a C-terminal GalNAc moiety that establishes interactions with the lectin domain-binding site (Figs 1 and 2a). The sugar moiety for the three above glycopeptides adopts a perpendicular conformation with respect to the peptide backbone. This also supports previous data suggesting that Cys residues bound to a GalNAc moiety mimic fairly well Thr residues linked to the same sugar^26^.

It is noteworthy that the C-terminal of MUC5AC-13, compared with the naked EA2 peptide, follows a divergent pathway towards its C-terminus (Fig. 2a,b). Both, MUC5AC-13 and EA2, bind in a competitive manner engaging potential acceptor sites close to the UDP (Fig. 2a,b). The different binding modes found for the peptides emphasize the plastic nature of the peptide-binding groove, which makes it potentially important to sample a large number of different acceptor substrates with multiple acceptor sites.

At the peptide-binding groove level, the specificity of GalNAc-T2 for the peptides MUC5AC-13 and EA2 (same interactions are also present for MUC5AC-Cys13 and MUC5AC-3-13) is governed mainly by hydrophobic interactions with Val255, Phe361, His365, Leu270, Phe377, Phe280, Trp282 and Tyr284 that are residues located in a solvent-exposed hydrophobic pocket (Fig. 2a,b). Both peptides are also tethered by hydrogen bonds with Trp282, His365 and Ser373 (Fig. 2a,b). Of four amino acids widely conserved among isoforms (Phe361, Phe280, Trp282 and Tyr396; Supplementary Fig. 1a), Phe361, Phe280 and Trp282 are key residues in the recognition of common peptide motifs such as Pro-x-Pro (where x is usually a small hydrophobic residue; Fig. 2a,b) found in acceptor substrates. In fact, site-directed mutagenesis of Phe361 and Trp282 to Ala residues almost eliminates the activity of the enzyme, and thus confirms the importance of these amino acids in peptide recognition (Fig. 2c and Supplementary Fig. 1b).

In all cases, the lectin domains appear to interact only with the GalNAc moiety of the glycopeptides without any discernable interaction with the peptide backbone (Fig. 2a). The lectin domains of GalNAc-Ts consist of three pseudo repeat regions termed α, β and γ, which each potentially may bind GalNAc (Supplementary Fig. 1c). However, it is accepted that the lectin domains from human GalNAc-T1 contain two functional GalNAc-binding sites (α and β), whereas GalNAc-T2 (α), GalNAc-T4 (α) and GalNAc-T10 (β) contain only one^21^. Unlike GalNAc-T10, in which the sugar moiety is located on the β-site^15^, the *C*-terminal GalNAc of the glycopeptides in GalNAc-T2 is located on the α-site of the lectin domain and is tethered by conserved residues such as Asp458, Asn479, Tyr471 and His474 (equivalent residues for β-binding site in GalNAc-T10 are Asp525, Asn544, Tyr536 and His539; Fig. 2a and Supplementary Fig. 1a). This explains why Asp458 (the only amino acid establishing two hydrogen bonds with the sugar moiety, Fig. 2a) is important for GalNAc-peptide substrate specificity and consequently in tuning the glycosylation profile of these enzymes^11^,^17^,^27^.

### The sugar nucleotide-binding site

**Figure 3:**
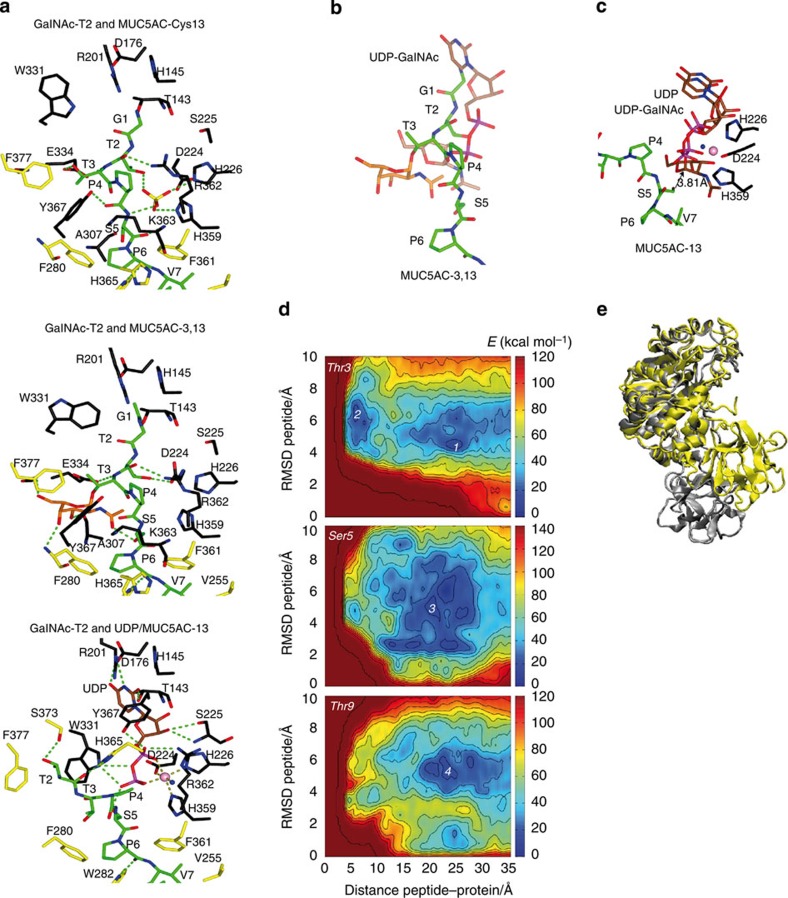
Structural features of the sugar nucleotide-binding site and metadynamics simulations of substrate binding. (**a**) Close-up view of the sugar nucleotide-binding site for GalNAc-T2-MUC5AC-Cys13, GalNAc-T2-MUC5AC-3-13 and GalNAc-T2-UDP-MUC5AC complexes. Hydrogen bond interactions and Mn^+2^ coordination are shown as dotted green and brown lines, respectively. A sulfate molecule is depicted in GalNAc-T2-MUC5AC-Cys13 complex. Peptides, UDP and amino acids are shown with the same colours as in Fig. 2. (**b**) Overlay of one of the monomers of GalNAc-T2 containing UDP-GalNAc (PDB entry 4D0T^16^) in the active site with GalNAc-T2-MUC5Ac-3-13 complex. The GalNAc moiety of UDP-GalNAc and MUC5AC-3-13 is shown as brown and orange carbon atoms, respectively. (**c**) Overlay of one of the monomers of GalNAc-T2 containing UDP-GalNAc (PDB entry 4D0T^16^) in the active site with GalNAc-T2-UDP-MUC5AC-13 complex. The structure shows that Ser5 is close to the anomeric carbon of UDP-GalNAc. (**d**) Free energy landscapes of peptide binding for each one of the three-acceptor sites. (**e**) Comparison between a reactive conformation of the peptide MUC5AC-13 for the Thr9 (yellow) and Thr3 (grey) acceptor sites.

Both the UDP and glycopeptides can bind to a versatile sugar nucleotide-binding site with a flexible loop adopting both open and closed conformations (Figs 1 and 3a). Although the *N*-terminal of MUC5AC-13 is covered by the flexible loop, the *N*-termini of MUC5AC-Cys13 and MUC5AC-3-13 are exposed to the solvent and inserted into the sugar nucleotide-binding site (Fig. 1c). The latter two glycopeptides adopt very similar binding modes, although some differences stand out towards the *N*-terminus (RMSD of 0.72 Å for aligned Cα atoms corresponding to residues Gly1-Ser5). The Gly1–Ser5 interactions with GalNAc-T2 are mainly governed by common hydrogen bonds with Glu334, Arg362 and Lys363, and distinct ones in particular for Asp224 and Tyr367 (Fig. 3a). There is an extra sulfate molecule in the GalNAc-T2-MUC5AC-Cys13 complex that occupies the same position found for the manganese atom in the competent structures such as the GalNAc-T2-MUC5AC-13-UDP complex (Fig. 3a; the interactions of GalNAc-T2 with UDP were discussed earlier)^14^,^16^. MUC5AC-Cys13 establishes further hydrogen bond interactions with this sulfate molecule (Fig. 3a). For the diglycosylated peptide, an unprecedented GalNAc-binding site, formed by aromatic residues such as Phe280, Tyr367 and Phe377, is found (Fig. 3a). This GalNAc moiety linked to Thr3 is tethered by Ser5, Phe280, Ala307, Gly333 and Phe377, and is found close to the sugar unit of UDP-GalNAc (average atomic shift of 5.29 Å; see Fig. 3a,b for further information). It is also important to emphasize that the lectin domain, through the interaction with the *C*-terminal GalNAc moiety of MUC5AC-13, imposes *N*-terminal residues of the glycopeptide close to UDP (Fig. 3a). Particularly, Ser5 is quite close to UDP (the distance between the Ser5 oxygen and the β-phosphate is 3.81 Å) and is potentially in a position to attack the anomeric carbon of UDP-GalNAc (see Fig. 3c for further information). This structure is compatible with the fact that GalNAc-T2 prefers to glycosylate *N*-terminal acceptor sites of glycopeptides efficiently, but the structure itself does not explain why Thr3 is the most favourable acceptor site, as shown earlier by kinetic studies, or why glycosylation optimally takes place at acceptor sites located ten residues *N*-terminal of an existing site of glycosylation^17^,^23^.

### Structure-guided metadynamics

In an attempt to answer why Thr3, positioned ten residues *N*-terminal to the prior glycosylated Thr13, is the most favourable acceptor site in comparison to less efficient acceptor sites such as Ser5 and Thr9, we modelled the binding of a glycopeptide to GalNAc-T2 using an enhanced sampling molecular dynamics (MD) approach (metadynamics)^28^. A ternary complex of GalNAc-T2 with UDP-GalNAc and a glycopeptide was used as the initial structure for the calculations. Room temperature MD simulations were performed to equilibrate the enzyme complex (see details in Supplementary Methods). Afterwards, the binding of the glycopeptide to the active site was monitored by metadynamics using two collective variables. The first variable was the distance between the centre of mass of the UDP-GalNAc and the centre of mass of the side chain of the peptide acceptor sites (Thr3, Ser5 or Thr9). This variable measures directly the penetration of the peptide into the active site. The second collective variable was taken as the RMSD of the peptide structure and measures changes in peptide conformation.

The free energy landscapes of peptide binding reconstructed from the metadynamics simulations show two main energy minima for Thr3 (minima **1** and **2** in Fig. 3d) at distances of 20–25 Å (outer minimum) and 4.5 Å (inner minimum), respectively, but only a broad outer minimum for Ser5 and Thr9 (**3** and **4**, respectively, in Fig. 3d). The outer minima (**1**, **3** and **4**) correspond to conformations in which the peptide is far from the active site and mainly interacts with the lectin domain (Supplementary Fig. 2a). Instead, the inner minimum (**2**, Thr3 glycosylation) corresponds to a reactive conformation, very close to the one found in the crystal structure of the GalNAc-T2-UDP-MUC5AC-13 complex (Supplementary Fig. 2a). Similar inner structures on the Ser5 and Thr9 maps are less stable than their corresponding outer global minimum, and thus much less populated. Consequently, the glycosylation reaction is expected to be less efficient for Ser5 and Thr9 than for Thr3.

The order of the preferred glycosylation site can be easily visualized by representing the free energy of the binding process versus the distance between the acceptor amino acid and the UDP-GalNAc donor (Supplementary Fig. 2b). It is important to note that only Thr3 glycosylation shows a deep inner minimum corresponding to reactive configurations.

A comparison of the protein structure for Thr9 and Thr3 glycosylation at short distances (Fig. 3e) shows that Thr9 glycosylation requires a highly compact and strained protein conformation in which the lectin domain approaches the catalytic domain. This conformation differs from the less compacted structure found in our crystal structure in complex with UDP-MUC5AC-13 (RMSD of 2.51 Å for 495 aligned Cα atoms). Notably, our simulation results are consistent with the previously reported solution studies, in which Thr3 was shown to be glycosylated preferentially over Ser5 and Thr9 (ref. 17), and also explains why GalNAc-T2 prefers to glycosylate *N*-terminal residues that are ten residues apart of a prior *C*-terminal glycosite. However, several questions arise on the molecular basis of how GalNAc-T2 achieves catalysis on residues close or very distant to an already attached GalNAc *O*-glycan, or whether the more compact structure shown above really exists in solution.

### GalNAc-T2 adopts compact and extended conformations

**Figure 4:**
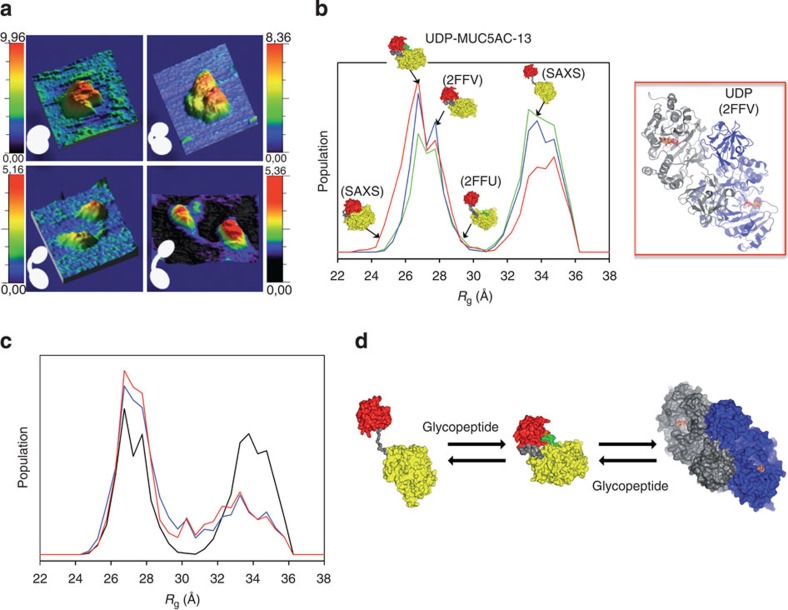
Three-dimensional topography AFM images of single molecules in different conformations and SAX analysis of GalNAc-T2. (**a**) (Top-left panel) A very compact structure image of the apo form showing an overlap of a domain on the other. The average height of these features is ≈9 nm. Compact image of the enzyme bound to UDP-GalNAc/Mn^+2^ and extended structures images of the enzyme bound to UDP/Mn^+2^ and EA2 are shown in the top-right and bottom panel, respectively. Small white figures representing different conformations of GalNAc-T2 are shown for clarification purposes (insert). (**b**) (Left panel) *R*_g_ distributions derived from the EOM analysis of monomeric GalNAc-T2 apo form at the three concentrations measured (2.5 (red), 5.0 (blue) and 10.0 (green) mg ml^−1^) identifying ensembles of monomeric compact and extended structures. Overall structures of GalNAc-T2 with different *R*_g_s are shown with lectin and catalytic domains in red and yellow, respectively, and flexible linker is shown in grey. It is noteworthy that our structure of GalNAc-T2-UDP-MUC5AC-13 complex is located in the left peak. (Right panel) Of the three crystallographic dimers tested, the SAXS data fit better with the dimer belonging to the PDB entry 2FFV^14^. (**c**) *R*_g_ distributions for the monomeric forms derived from the EOM analysis displaying the decrease of the relative population of the extended conformation upon addition of the glycopeptide. GalNAc-T2 is at a fixed concentration of 5 mg ml^−1^ (black is for the enzyme without the peptide) with increasing amounts of the MUC5AC-13 peptide (1 (blue) and 2 mM (red) of the MUC5AC-13 peptide. (**d**) GalNAc-T2 is in equilibrium with an ensemble of compact and extended structures, and dimeric forms. This equilibrium is partly shifted towards the ensemble of compact structures in the presence of peptides and mainly glycopeptides.

To understand the conformational state of GalNAc-T2 under ligand-binding and -unbinding conditions, we applied initially time-lapse AFM experiments. A series of AFM topography images were recorded of single GalNAc-T2 molecules under different conditions and in three distinguishable conformational states: (i) a highly compact structure similar to the one above found through molecular modelling (Figs 3e and 4a, top-left panel) and in which the two domains interact closely even though a slot is still observed between them; (ii) a compact structure similar to our crystal structures that displays a clear slot (Fig. 4a, top-right panel and Supplementary Fig. 3a) and (iii) extended structures exhibiting fully separated domains (Fig. 4a, bottom panel and Supplementary Fig. 3b). Our AFM experiments (Fig. 4a, bottom panel and Supplementary Fig. 3b) support, for the first time, that the extended state visualized in the crystal structure of GalNAc-T2 in complex with UDP and the EA2 peptide (PDB entry 2FFU^14^; Fig. 1c), in which the domains are separate, is not an artefact of the crystallographic conditions.

The highly compact and compact species show height values of ≈8–10 nm (Fig. 4a, top panel), whereas the average height for the extended species is smaller, reaching ≈5 nm (Fig. 4a, bottom panel). The size of the separated domains in the images is barely larger than that estimated from the crystal structure (3.5 and 4.7 nm for the lectin and the catalytic domains, respectively, in PDB entry 2FFU^14^) mainly as a result of hydration^29^. The intermediate sizes observed for the compact species thus suggest a slight overlap between both domains of the enzyme.

The enzyme in the apo form or bound to UDP or UDP-GalNAc in the presence of Mn^+2^ adopts either the very compact or the compact structure (Fig. 4a, top panel). However, the three different conformational states are present when the enzyme is either bound to EA2 or to glycopeptides such as MUC5ACs-Cys9 (see sequence in Supplementary Table 1) or MUC5AC-13 alone or in combination with UDP/Mn^+2^ (Fig. 4a). Although our data does not provide relevant information of the distribution between different conformational states, clearly demonstrates the highly dynamic nature of the GalNAc-T2 enzyme.

### GalNAc-T2 monomeric compact conformations

To quantify the GalNAc-T2 conformational states in solution, we measured SAXS data for a large number of conditions. The capacity to describe this data with available crystallographic structures for the monomeric and dimeric forms of GalNAc-T2 was tested with the programme CRYSOL^30^. The poor agreement of either structures to the experimental SAXS curves (see Supplementary Table 3), prompted us to study the enzyme flexibility using the Ensemble Optimization Method (EOM)^31^. Ensemble analysis of solution scattering curves for the apo enzyme at different concentrations revealed a unique distinguishable bimodal distribution of monomeric compact and extended structural ensembles that have not been found before in other types of proteins by using this method (Fig. 4b and Supplementary Figs 3c, 4 and 5). In addition, the ensemble analysis identified compact dimers that reached up to ≈40% at the highest concentration measured (10 mg ml^−1^; Supplementary Table 3). Analysis of the data shows that both monomeric conformation ensembles are similarly populated but do not display a continuum of conformational states. The radius of gyration (*R*_*g*_) ranges between *R*_*g*_≈24–29.5 and ≈31–36 Å for the compact and extended structures, respectively, with negligible population in between (Fig. 4b). As already mentioned, a small percentage of potential structures with *R*_*g*_ between ≈29.5 and 31 Å are not displayed because they are likely insufficiently stable to be detected (Fig. 4b). These data reinforce the GalNAc-T2 dynamics inferred from our AFM experiments and demonstrates that GalNAc-T2 is even more flexible than shown by AFM experiments. In contrast to AFM, SAXS is also able to identify a significant population of dimers that supports the dimeric arrangement found for our crystal structures, with the exception of the PDB entry 2FFU^14^, in which the enzyme is monomeric (we used the PISA server to determine the quaternary structure of GalNAc-T2 in the crystals). The SAXS fits with EOM were notably better when the three crystallographic dimeric arrangements were combined with the monomeric forms (Fig. 4b, Supplementary Figs 3c, 4 and 5, and Supplementary Methods). In particular, three different pools were built consisting in 10,000 monomeric conformations that were separately enriched with 100 copies of the theoretical curves of each of the three dimeric arrangements tested (see Supplementary Methods). The best fit was achieved when the most compact dimer that possesses a buried area of 1,689 Å^2^ (PDB entry 2FFV^14^) was used. Dimers visualized in crystal structures with a tetragonal space group appear to be the most flexible compact structures of all dimers with ≈750 Å^2^ of buried area. This latter arrangement may explain why this particular dimer can bind glycopeptides. In all three cases, the dimers display similar contacts between the lectin domains and the lectin of one monomer with the catalytic domain of a second monomer (Fig. 4b and Supplementary Fig. 3c).

Notably, the GalNAc-T2-UDP-MUC5AC-13 complex with an *R*_*g*_ of 26.41 Å is one of the most populated compact molecules in solution unlike less representative structures such as the one found for the PDB entry 2FFU^14^ with *R*_g_ of 30.5 Å (Fig. 4b). This result exemplifies that GalNAc-T2 preferentially adopts conformations that might be important for catalytic aspects of this enzyme.

Further SAXS experiments were carried out in the presence of the ligands, which provided very intriguing results (Supplementary Table 3). The enzyme either without ligands or complexed with UDP or UDP-GalNAc shows a higher percentage of dimers (28–44%) relative to the enzyme in complex with glycopeptides, naked peptides or peptides-UDP (0–28%; Supplementary Table 3). In particular, dimers almost disappear from solution when GalNAc-T2 at a fixed concentration of 5 mg ml^−1^ is incubated with increasing concentrations of Muc5AC-13 (Supplementary Table 3 and Supplementary Figs 3d and 5). Moreover, similar amounts of compact and extended monomeric ensembles exist for the enzyme without ligands or complexed with UDP or UDP-GalNAc; although in some cases, a slight increase in the amount of extended conformations occurs (Supplementary Table 3). This equilibrium is partly shifted to compact monomeric forms in the presence of naked peptides and significantly shifted in the presence of glycopeptides alone or with UDP (reaching values of 59–70% in the presence of peptides; Fig. 4c and Supplementary Table 3). Overall, these results indicate a direct association between the distribution of GalNAc-T2 conformation states and acceptor substrate binding with catalysis, and consequently, the glycosylation profile of GalNAc-T2 (Fig. 4d). In particular, an increase of the monomeric compact structures ensemble appear to be required for catalysis on glycopeptides.

### The role of the flexible linker

**Figure 5:**
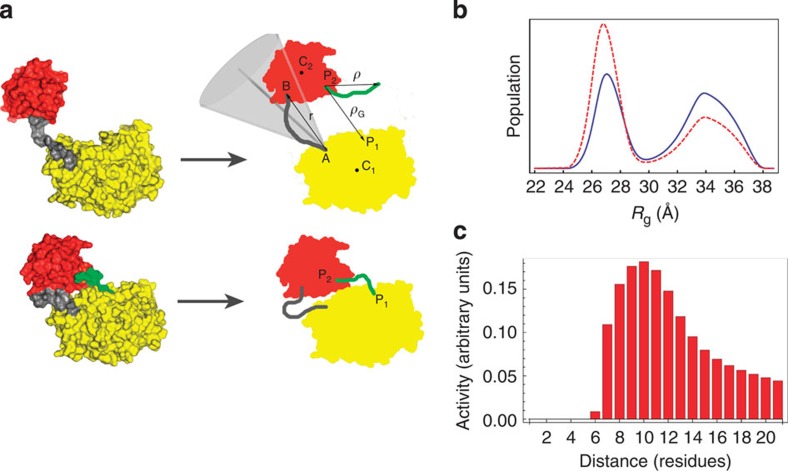
Coarse-grained theoretical model of GalNAc-T2. (**a**) The crystal structure of GalNAc-T2-UDP-MUC5AC-13 (bottom left) is taken as a reference for the model (bottom, right). The apo form, without the peptide (green), is obtained by removing the latter from the coordinates file. Upon fixing the relative orientation of the two domains (red and yellow for the lectin and catalytic domains, respectively), protein conformations depend on the flexible linker (grey), modelled as a (non-extendable) semi-flexible chain with an attractive interaction between its ends. All other conformations of the protein (top left) are modelled on the basis of the previous one, by letting the end-to-end vector (*r*) explore the region within a cone (light grey) of angular amplitude *2θ*_0_ (see Supplementary Methods for details). In the holo-form, the peptide (green) is modelled as a WLC of length (*l*) residues (the distance between the glycosylating sites on the ligand), bound at P_2_ on the lectin domain. The enzymatic reaction can take place in any protein conformation providing that the ligand free-end finds correct place P_1_ on the catalytic domain. (**b**) Model predictions for the probability distribution [*G(R*_*g*_)] of the radius of gyration *R*_g_. The apo-form is shown as a blue solid line, whereas the holo-form complexed with peptide is shown as a red dashed line. (**c**) Enzymatic activity [*σ*(*l,lc*)] as a function of the residue separation (*l*) between potential glycosites and a prior fixed glycosite of the glycopeptides (see Supplementary Methods).

To further understand the unique dynamics of GalNAc-T2 and the consequences for catalysis, we developed a simple computational model in which the protein is treated as two interacting rigid domains, connected by a flexible linker that is described as a worm-like-chain (WLC model is used to describe the behaviour of semi-flexible polymers; Fig. 5a)^32^. The model predicts, at a qualitative level, the bimodal nature of the *R*_g_ distribution revealed by SAXS data (Fig. 5b).

Within this model, the peak at high values of the *R*_g_ (*R*_g_=31–37 Å) is associated with the equilibrium distribution of the WLC, whereas the peak at low values of the *R*_g_ (*R*_g_=24.5–29.5 Å) corresponds to the interaction of both domains (Fig. 5b and Supplementary Fig. 6). In this context, the addition of peptides is expected to increase the effective interdomain interaction by increasing the interaction surface. Accordingly, if we strengthen the interaction energy (*ɛ*; see Supplementary Methods), we can reproduce the increase of the peak at lower *R*_g_ (Fig. 5b).

Importantly, this simple model can shed light on the different catalytic activity of GalNAc-T2 on glycopeptide substrates in which multiple acceptor sites are found at different distances from a GalNAc *O*-glycan at a fixed position (Fig. 5c). The crystal structure of the GalNAc-T2-UDP-MUC5AC-13 complex suggests that the GalNAc moiety of a glycopeptide substrate may first bind to the lectin domain, which leads to enhance the probability that GalNAc-T2 transfers another GalNAc residue to an acceptor site approximately ten residues N-terminal of the first GalNAc residue located at the lectin domain-binding site. This rationale may help to explain previous work, in which the catalytic efficiency of GalNAc-T2 is higher with glycopeptides than with naked peptides that only contain one acceptor site^23^.

Despite the fact that the catalytic process is intrinsically not one at equilibrium, the equilibrium behaviour of our simple model grasps the fundamental aspects of the enzymatic activity. By keeping the first glycosylated site bound to the lectin domain at point P2 (Fig. 5a), only the probability that the acceptor site of the peptide (also described as a WLC) is found in the correct position P1 at the active site is taken into account (see Methods and Supplementary Methods). Our computational model reasonably reproduces not only the previously reported broad glycosylation profile of GalNAc-T2, but also the peak where a maximum in the enzymatic activity is achieved, which in turn corresponds to an acceptor site ten residues apart from a prior site of glycosylation^23^ (Fig. 5c).

The flexible linker is a unique feature of the GalNAc-T isoenzymes whose motion is responsible for the GalNAc-T2 dynamics, and consequently, the glycopeptide glycosylation capacity of GalNAc-T2. The flexible linker is such an important structural feature that if we computationally fix it in its position in the crystal structure, the glycosylation activity profile changes and the predicted activity decreases significantly (≈5,000-fold reduction; Supplementary Fig. 7).

In summary, the glycosylation activity profile of GalNAc-T2 is directly coupled to the flexible linker motion that dictates the unique dynamics of GalNAc-T2, and to the GalNAc-T2 binding to glycopeptides that increases the population of compact structures. This model, which is independent of whether the glycopeptides have prior GalNAc *O*-glycans *N*- or *C*-terminal to available to acceptor sites, might help to explain the glycosylation profile of other GalNAc-Ts isoforms.

## Discussion

In the present study, we have determined the first crystal structures of GalNAc-T2 in complex with defined GalNAc-glycopeptide substrates. These structures in combination with AFM and SAXS experiments, as well as with theoretical simulations, reveal how GalNAc-T2 selectively glycosylates unused acceptor sites located in the *N*-terminal and optimally ten residues apart from a prior GalNAc glycan. Our results show that GalNAc-T2 populates an ensemble of compact and extended monomeric structures, as well as dimeric ones. In the presence of the acceptor substrates, the dimers disappear while the compact monomeric structures get more populated than the extended one, thus pointing at a prominent role of compact structures for enzymatic activity. Although flexibility in an enzyme is hardly surprising, the picture emerging from our results suggests a peculiar role of the flexible linker, which acts as a conformational dial to foster glycosylation of substrate peptides with different distances between sites of sequential glycosylation. We demonstrate that the lectin domain, which is a unique feature of the large GalNAc-T isoenzyme family, modulates the catalytic functions and coordinates the follow-up order of glycosylation of protein substrates.

In contrast to the role of the GHs CBMs, GalNAc-Ts lectin domains are not only important to bind to the sugar moiety of glycopeptides, but also to guide catalysis to other unused acceptor sites. This study provides further support for the general model that we proposed for GalNAc-*O*-glycosylation, where a number of GalNAc-T isoenzymes orchestrate *O*-glycosylation in a coordinated manner before and in competition with subsequent elongation of *O*-glycans^2^. In addition, our analysis affords the first experimental evidence of the molecular mechanism of an exquisite additional level of control of *O*-glycosylation. Moreover, the results from the coarse-grained model suggest that the basic features, responsible for flexibility and for the activity profiles, do not depend on the sequence details, so that the proposed model might apply to other GalNAc-T isoenzymes and help explain their dynamics and activity profiles.

Together, these findings may guide the rational design of mechanism-based modulators for this relevant family of enzymes. The finding of inactive and active conformational states in GalNAc-Ts together with the motion of the flexible loop and its association with activity somehow resemble similar features found for the large family of protein kinases. In general, similar states and an activation flexible loop, marked by conserved DFG and APE motifs, are also found in protein kinases and these structural features have been exploited to discover protein kinase inhibitors^33^. We believe that modulators for this particular family of GalNAc-Ts can be developed using similar approaches that will also include the targeting of the lectin domains.

## Methods

### Cloning, site-directed mutagenesis and purification

The expression plasmid pPICZαA*galnact2* (K75-Q571), previously described^16^, was used as a template for introducing the following single amino-acid changes by site-directed mutagenesis as follows: Trp282Ala and Phe361Ala. Site-directed mutagenesis was carried out following the QuikChange protocol (Stratagene), using the Phusion Hot Start II High-Fidelity DNA Polymerase (Thermo Scientific). All plasmids were verified by sequencing (Sistemas Genómicos, Valencia, Spain). The mutants were purified using the purification protocol of the wild-type enzyme described previously^16^.

### Crystallization

In all cases, crystals were grown by hanging drop vapour diffusion at 18 °C. Crystals of the inactive GalNAc-T2 form in complex with the peptide MUC5AC-Cys13 were obtained by mixing 2 μl of protein solution (a mix formed by 7 mg ml^−1^ GalNAcT-2, 5 mM UDP, 5 mM MnCl_2_, 5 mM MUC5AC-Cys13 peptide in 25 mM Tris (pH 8.0), 0.5 mM EDTA and 1 mM tris(2-carboxyethyl)phosphine (TCEP)) with 2 μl of precipitant solution (25% PEG 4,000, 400 mM ammonium sulfate and 100 mM sodium citrate pH 6) against 500 μl of precipitant solution. Crystals of the inactive GalNAc-T2 form in complex with the peptide MUC5AC-3-13 were obtained by mixing 2 μl of protein solution (a mix formed by 7 mg ml^−1^ GalNAcT-2, 5 mM UDP, 5 mM MnCl_2_, 6 mM MUC5AC-3–13 peptide in the same buffer described above) with 2 μl of precipitant solution (1.6 M ammonium sulfate and 100 mM Tris, pH 9) and equilibrated against 500 μl of precipitant solution. Crystals of the GalNAc-T2 active form in complex with UDP/Mn^+2^ and the peptide MUC5AC-13 were obtained in similar conditions as the inactive forms. The protein solution consisting of 3.5 mg ml^−1^ of GalNAcT-2, 5 mM UDP, 5 mM MnCl_2_ and 6 mM MUC5AC-13 in the same buffer described above was mixed with the precipitant solution containing 1.6 M ammonium sulfate, 100 mM sodium chloride and 100 mM HEPES (pH 7). The crystals were cryoprotected in saturated lithium sulfate and frozen in a nitrogen gas stream cooled to 100 K.

### Structure determination and refinement

All data were processed and scaled using the XDS package^34^ and CCP4 software^35^. Relevant statistics are given in Table 1. The crystal structures were solved by molecular replacement with Phaser^35^ and using the PDB entry 4D0T as the template. Initial phases were further improved by cycles of manual model building in Coot^36^ and refinement with REFMAC5 (ref. 37). The final models were validated with PROCHECK^38^ and model statistics are given in Table 1. The AUs of the tetragonal crystals contain 1 molecule of GalNAc-T2 (Fig. 1). Coordinates and structure factors have been deposited in the Worldwide Protein Data Bank (wwPDB, and see Table 1 for the pdb codes).

### Synthesis of peptides and glycopeptides

All peptides were synthesized by stepwise solid-phase peptide synthesis using the Fmoc strategy on Rink Amide MBHA resin (0.1 mmol). *O*-α-D-GlcNAc-L-Thr^39^ or *S*-α-D-GlcNAc-L-Cys^26^ building blocks (2 equiv.) were prepared as described in the literature and manually coupled. The other Fmoc amino acids were coupled in the automated mode in an Applied Biosystems 433A peptide synthesizer using 10 equiv. and HBTU as a coupling agent. The *O*-acetyl groups of the sugar moiety were deprotected in a mixture of NH_2_NH_2_/MeOH (7:3). The derivatives were then released from the resin, and all acid-sensitive side-chain-protecting groups simultaneously removed using 95% trifluoroacetic acid (TFA), 2.5% triisopropylsilane (TIS) and 2.5% H_2_O, followed by precipitation with diethyl ether. Finally, all the compounds were purified by HPLC on a Waters Delta Prep chromatograph (Phenomenex Luna C18(2) column (10 μ, 21.20 × 250 mm^2^)). Additional details are in Supplementary Methods.

### Tryptophan fluorescence spectroscopy

Fluorescence spectroscopy was used to determine the dissociation constants of GalNAc-T2 against the peptides MUC5AC, MUC5AC-13 and MUC5AC-3-13 (ref. 40).

All experiments were carried out in a Cary Eclipse spectrofluorometer (Varian) at 25 °C with GalNAc-T2 at 1 μM, and concentrations of peptides varying from 1 to 500 μM in 25 mM Tris, 150 mM NaCl, pH 7.5. The same experiments were also performed in the same buffer but containing 400 μM UDP and 1 mM MnCl_2_. Fluorescence emission spectra were recorded in the 300–400 nm range with an excitation wavelength of 280 nm, with slit width of 5 nm. The data analysis was performed in Prism (GraphPad software) considering a model with a single binding site (see equation (1), where F0 is the intrinsic fluorescence of the enzyme in the absence of quencher (*Q*), F1 is the observed fluorescence at a given quencher concentration, *f*_a_ is the fractional degree of fluorescence and *K*_d_ is the dissociation constant.


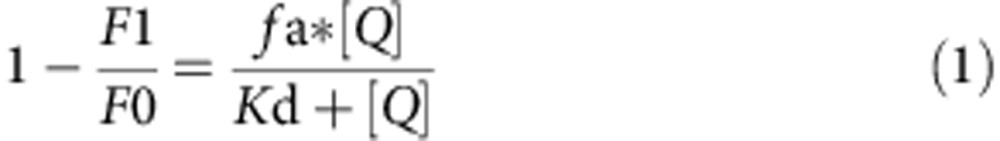


### Analysis of the enzymatic reaction by MALDI-TOF-MS

We carried out mass spectrometry analyses to determine the transfer activity of GalNAc-T2 apo form and mutants using UDP-GalNAc and the MUC5AC and MUC5AC-13 acceptor peptides. *In vitro* glycosylation assays were performed as product development assays in 50 μl buffer (25 mM cacodylic acid sodium, pH 7.4, 10 mM MnCl_2_, 0.25% Triton X-100), 5 mM UDP-GalNAc (Sigma), 0.2 mM of acceptor peptides and 0.4 μM of purified GalNAc-T2 wild-type or mutant enzymes at 37 °C. For time-course evaluation, 2 μl of the reaction mixtures were taken at 10 min, 20 min, 40 min, 1 h, 2 h, 4 h, 20 h and 44 h (2 mM of UDP-GalNAc and 0.2 μM of enzyme were added into the above mixture after 20 h reaction to push the reaction for another 24 h), and analysed by MALDI-TOF-MS. Evaluation of incorporation of GalNAc residues into peptide substrates was performed by matrix-assisted laser desorption/ionization–time of flight-mass spectrometry (MALDI-TOF-MS). 2 μl of reaction mixtures were diluted with 18 μl of 0.1% TFA/H_2_O, and 1 μl mixed with 1 μl of 10 mg ml^−1^ 2,5-dihydrobenzoic acid dissolved in ACN/H_2_O (7:3). Acquisition of MS spectra was performed on MALDI-TOF instrument, Bruker Autoflex using Bruker FlexControl 3.4 software. Spectra were recorded in the positive ion mode and the raw spectra were processed by Bruker FlexAnalysis 3.4 software. The specific activities under linear conditions were inferred from the mass spectrometry data. One unit of enzyme is defined as the amount of enzyme that transfers 1 μmol GalNAc in 1 min using the standard reaction mixture.

### Computational details

MD simulations of the enzyme were performed with the Amber11 software package. The protein was modelled with the FF99SB force field, whereas all carbohydrate molecules were modelled with the GLYCAM06 force field^41^. A snapshot of the equilibrium MD simulation was taken for the metadynamics simulations. Additional details are in Supplementary Methods.

### AFM imaging

AFM measurements were performed using a MultiMode 8 AFM system (Bruker). Images were taken using the Tapping Mode with V-shaped silicon nitride cantilevers with integrated pyramidal 2 nm ultrasharp tips exhibiting a spring constant and a frequency of 0.03 N m^−1^ and 15 kHz, respectively (MSNL-D; Bruker Probes). Additional details are in Supplementary Methods.

### SAXS and data analysis

SAXS measurements were performed at the European Molecular Biology Laboratory on the storage ring PETRA-III (DESY-Hamburg) on the P12 beamline equipped with a robotic sample changer and a PILATUS-2M. The analysis of the SAXS data was performed by using the EOM^31^. Additional details are in Supplementary Methods.

### Coarse-grained model

We used as a template the crystal structure of the GalNAc-T2-UDP-MUC5AC-13 complex to generate our coarse-grained model. The protein was treated as two interacting rigid domains (the catalytic and the lectin domains) joined by a 12-residue semi-flexible linker that is described by a semi-elastic WLC model^32^,^42^,^43^ (Fig. 5a). We calculated the equilibrium canonical (Boltzmann) distribution of the end-to-end vector of the linker, as well as that of the glycopeptide, also considered as a WLC. The probability distribution of the *R*_g_ was derived from that of the end-to-end vector. All calculations were performed with Wolfram's Mathematica 9.0. Additional details are in Supplementary Methods.

## Author contributions

R.H.-G. designed the crystallization construct and solved the crystal structures. E.L.-N., M.R. and R.H.-G. purified the enzymes, crystallized the different complexes and refined the crystal structures. I.C., F.C., G.J.L.B. and J.M.P. synthetized the glycopeptides. M.C.P. and A.L. performed the AFM studies. E.L.-N. and P.Be. performed the SAXS experiments. P.Br. performed the coarse-grained model. J.I.-F. and C.R. performed the metadynamics simulations. Y.K. and H.C. perfomed the analysis of the enzymatic reaction by MALDI-TOF MS. R.H.-G. wrote the article with the main contribution of F.C., G.J.L.B., H.C., C.R., P.Br, P.Be. and A.L. All authors read and approved the final manuscript.

## Additional information

**Accession codes:** Coordinates and structure factors have been deposited in the Worldwide Protein Data Bank (wwPDB) with accession codes 5AJN, 5AJO and 5AJP.

**How to cite this article:** Lira-Navarrete, E. *et al*. Dynamic interplay between catalytic and lectin domains of GalNAc-transferases modulates protein *O*-glycosylation. *Nat. Commun.* 6:6937 doi: 10.1038/ncomms7937 (2015).

## Supplementary Material

Supplementary Information Supplementary Figures 1-7, Supplementary Tables 1-3, Supplementary Methods and Supplementary References

## Figures and Tables

**Table 1:** Data collection and refinement statistics*.

	**GalNAc-T2 -MUC5AC-Cys13**	**GalNAc-T2-MUC5AC-3,13**	**GalNAc-T2-MUC5AC-13-UDP-Mn**^**+2**^
*Data collection*			
Space group	I 4_1_	I 4_1_	I 4_1_
Cell dimensions			
*a*, *b*, *c* (Å)	87.35, 87.35, 178.43	87.2, 87.2, 179.07	87.27, 87.27, 178.59
*α*, *β*, *γ* (°)	90.0, 90.0, 90.0	90.0, 90.0, 90.0	90.0, 90.0, 90.0
Resolution (Å)	20–1.67 (1.76–1.67)	20–1.48 (1.56–1.48)	20–1.65 (1.74–1.65)
*R*_merge_†	0.103 (0.653)	0.058 (0.535)	0.043 (0.601)
*I*/σ(*I*)	11.3 (3.0)	18.6 (2.3)	22.1 (2.9)
Completeness (%)	99.8 (100)	99.6 (97.5)	99.8 (99.2)
Multiplicity	8.9 (9.2)	8.6 (4.2)	6.7 (5.8)
			
*Refinement*			
Resolution (Å)	20–1.67 (1.76–1.67)	20–1.48 (1.56–1.48)	20–1.65 (1.74–1.65)
No. of reflections (test)	76,947 (2,308)	110,205 (3,306)	79,709 (2,391)
*R*_work_/*R*_free_‡	0.196/0.232	0.149/0.178	0.156/0.185
*No. of atoms*			
Protein	4,023	4,040	3,991
Glycopeptide	114	129	107
Sulfate	60	155	110
Ethylenglycol	56	—	—
UDP	—	—	25
Mn^+2^	—	—	1
Water	556	800	614
*B-factors (Å*^*2*^)			
Protein	22.41	19.03	26.71
Glycopeptide	33.81	19.46	19.29
Sulfate	56.01	61.48	69.44
Ethylenglycol	38.75	—	—
UDP	—	—	21.88
Mn^+2^	—	—	19.29
Water	33.51	35.06	39.02
*R.m.s deviations*			
Bond lengths (Å)	0.019	0.012	0.013
Bond angles (°)	2.027	1.613	1.627
PDB ID	5ajn	5ajo	5ajp

UDP, uridine diphosphate.

^*^Highest resolution shell is shown in parenthesis.

^†^*R*_merge_=ΣhklΣ*i* |*I*(hkl)*i*—[*I*(hkl)]|/Σhkl Σ*i I*(hkl).

^‡^*R*_work_*=*Σhkl|*F*(hkl)_o_—[*F*(hkl)_c_]|/Σhkl *F*(hkl)_o_; *R*_free_ was calculated as *R*_work_, where *F*(hkl)_o_ values were taken from 3% of data not included in the refinement.
