# Supplementary material for: Dynamic interplay between catalytic and lectin domains of GalNAc-transferases modulates protein O-glycosylation
Source: Nat Commun. 2015 May 5;6:6937. doi: 10.1038/ncomms7937 (PMC4432651; doi:10.1038/ncomms7937)
Supplement: Supplementary Information — Supplementary Figures 1-7, Supplementary Tables 1-3, Supplementary Methods and Supplementary References [file ncomms7937-s1.pdf]

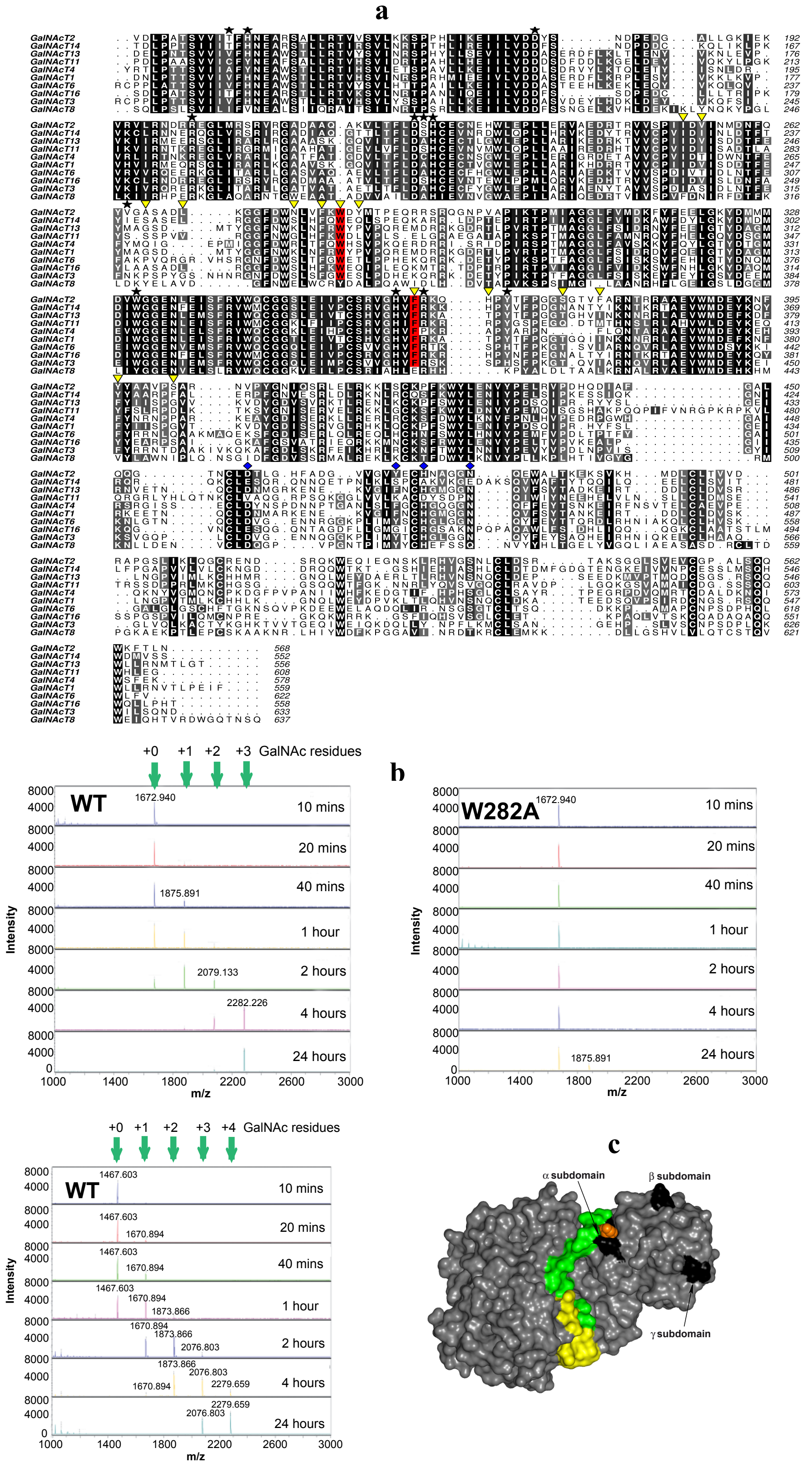

Supplementary Figure 1. (**a**) Sequence alignment of GalNAc-T2 (residues 133-568) with other GalNAc-Ts isoforms. Conserved residues are highlighted in black and similar residues are in grey. Residues forming the sugar-nucleotide, peptide and lectin domain binding sites are indicated as black stars, yellow inverted triangles and blue diamonds, respectively. Mutated residues are highlighted in red. (**b**) Time-course of the enzymatic reaction of the wild type enzyme and the mutant W282A with UDP-GalNAc and the MUC5AC-13 peptide followed by MALDI-TOF MS (Top panel). Time-course of the enzymatic reaction of the wild type enzyme with UDP-GalNAc and the MUC5AC peptide (Bottom panel). Green arrows indicate the MUC5AC-13 and MUC5AC peptides and the glycosylated forms, respectively. **(c)** Surface representation of GalNAc-T2 in complex with UDP and MUC5AC-13. Colors are indicated as **Fig. 1c** except for black colors that represent residues located in the different subdomains of the lectin domain**.**

a

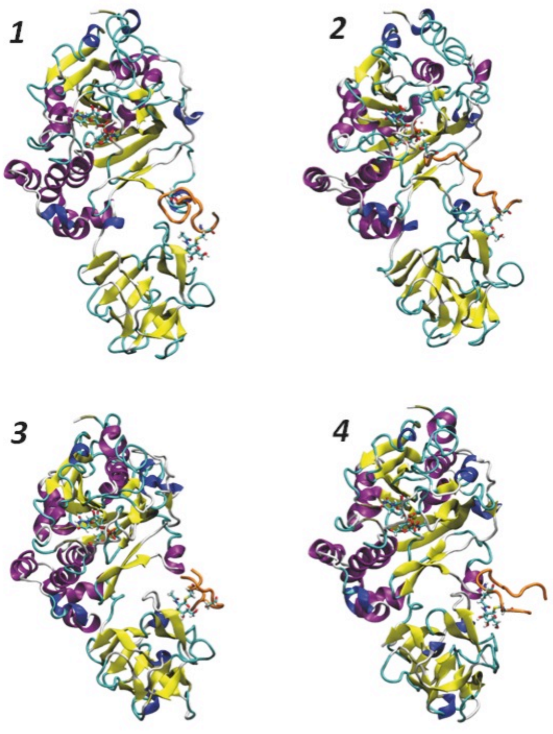

b

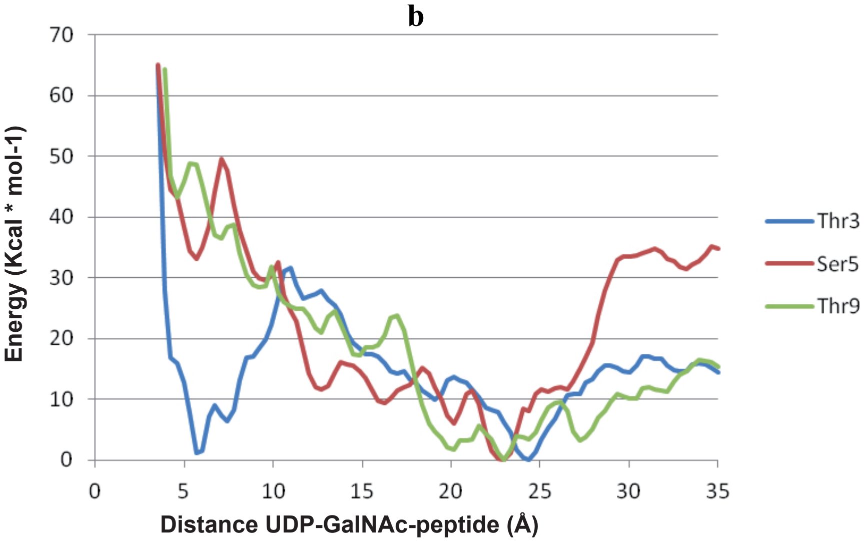

Supplementary Figure 2. (**a**) Representative conformations extracted from the metadynamics simulations. Conformations **1-4** correspond to those indicated in **Fig. 3d**. (**b**) Free energy of glycopeptide binding *versus* the distance between the acceptor amino acid and the UDP-GalNAc donor. The energy profile has been obtained by integrating the FEL with respect to the RMSD collective variable. It is clear that Thr3 is the unique glycosylation site with an energy minimum at short distances (approximately 5 Å), whereas the other acceptor sites present an energy minimum at higher distances.

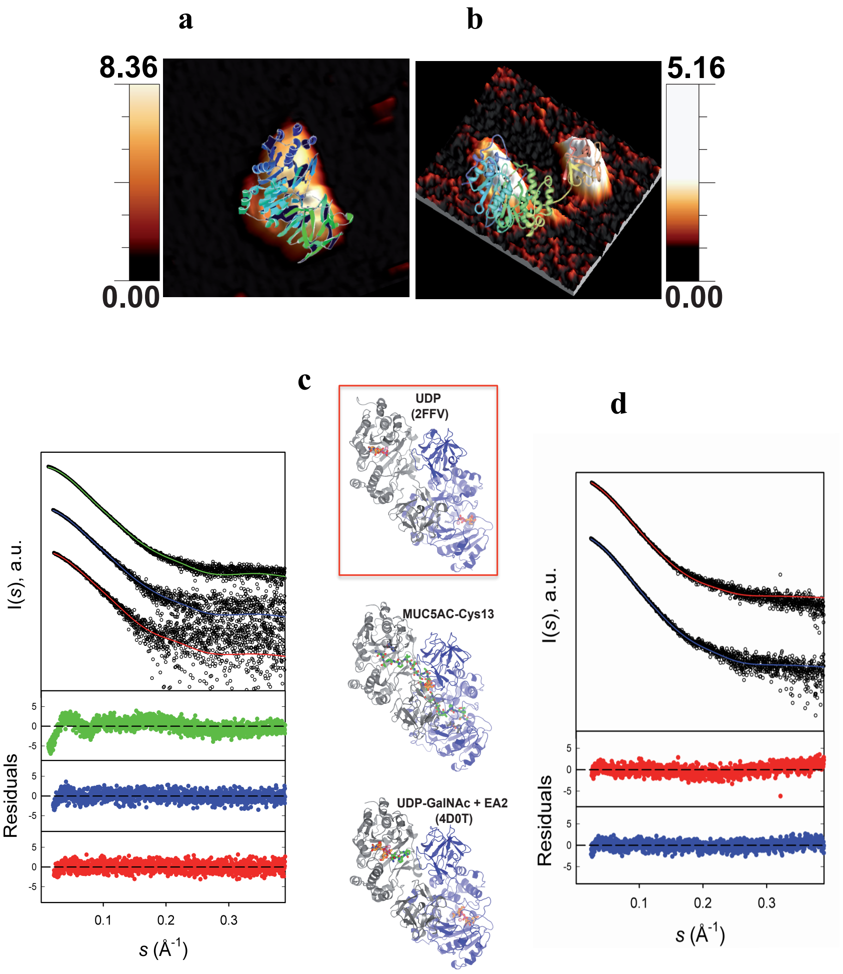

Supplementary Figure 3. (a) Overlay of the monomeric form of the GalNAc-T2-UDP complex (PDB entry 2FFV) on the 3D AFM topography image of a compact structure found in the presence of UDP-MUC5AC-13, and (**b**) overlay of GalNAc-T2-UDP-EA2 complex (PDB entry 2FFU) on the 3D AFM topography image of an extended molecule complexed with EA2 and UDP. (**c**) (Left panel) SAXS intensity profiles, [*I(s)*], as a function of the momentum transfer, (*s*), measured for GalNAc-T2 apo form at three concentrations (empty dots). EOM fits of the three profiles are displayed as solid lines for the 2.5 (red), 5.0 (blue) and 10.0 (green) mg/ml. Profiles have been displaced along the *I(s)* axis for clarity. The point-by-point residuals (same color-code) that indicate the high quality of the fit in the complete range of momentum transfer. (Right panel) Of the three crystallographic dimers tested, the SAXS data fits better with the dimer belonging to the PDB entry 2FFV. (**d**), SAXS intensity profiles [*I(s)*] as a function of the momentum transfer (*s*) for GalNAc-T2 at a fixed concentration of 5 mg/ml with increasing amounts of the MUC5AC-13 peptide. EOM fits are displayed as solid lines for the 1 (blue) and 2 mM (red) of the MUC5AC-13 peptide. The quality of the fit is shown in the point-by-point residuals displayed below with the same color-code.

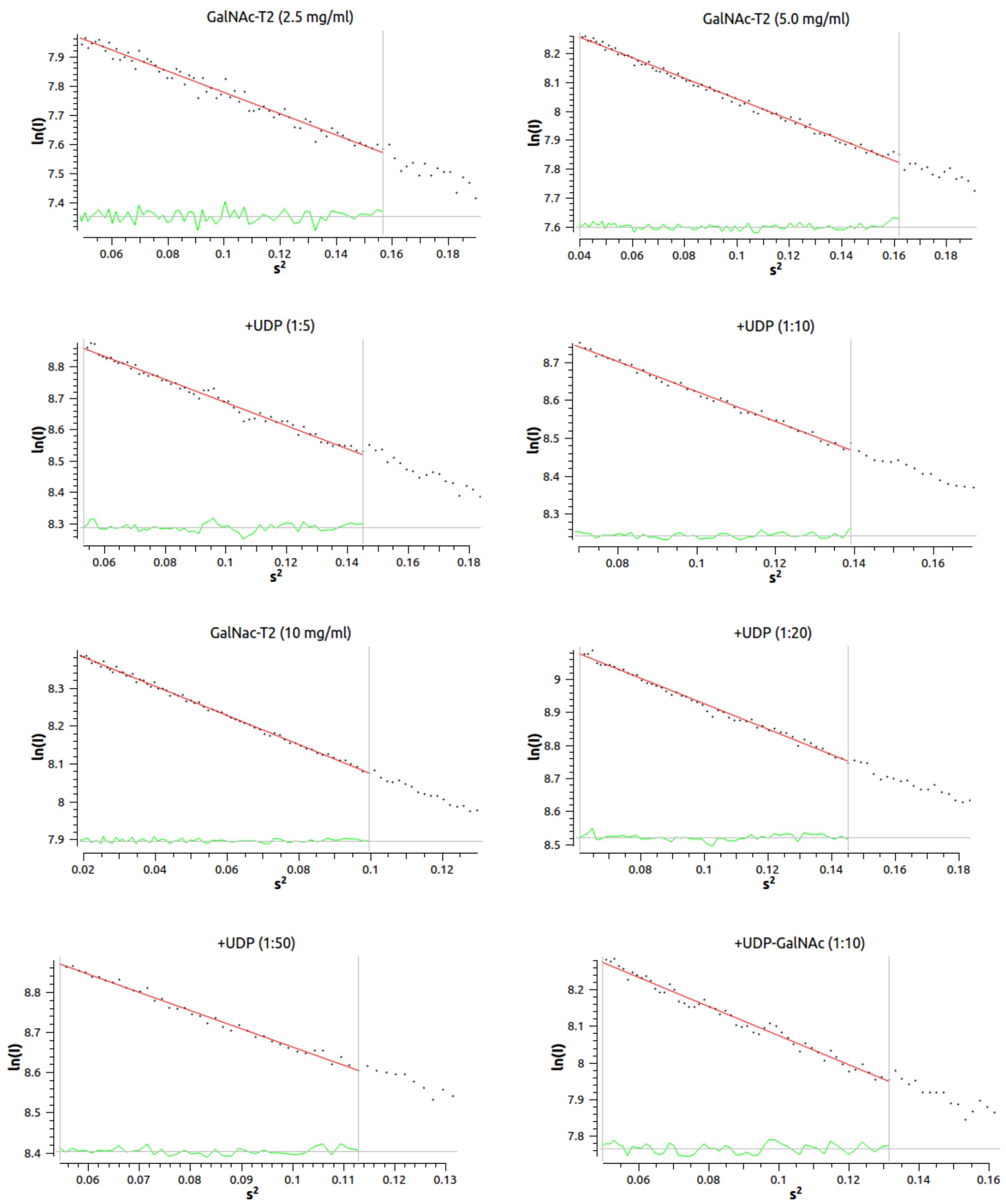

Supplementary Figure 4. Guinier's fits corresponding to samples described in **Supplementary Table 3**. In all the cases, an excellent linear fit is obtained allowing to derive precise radius of gyration, Rg, and forward scattering values, I(0).

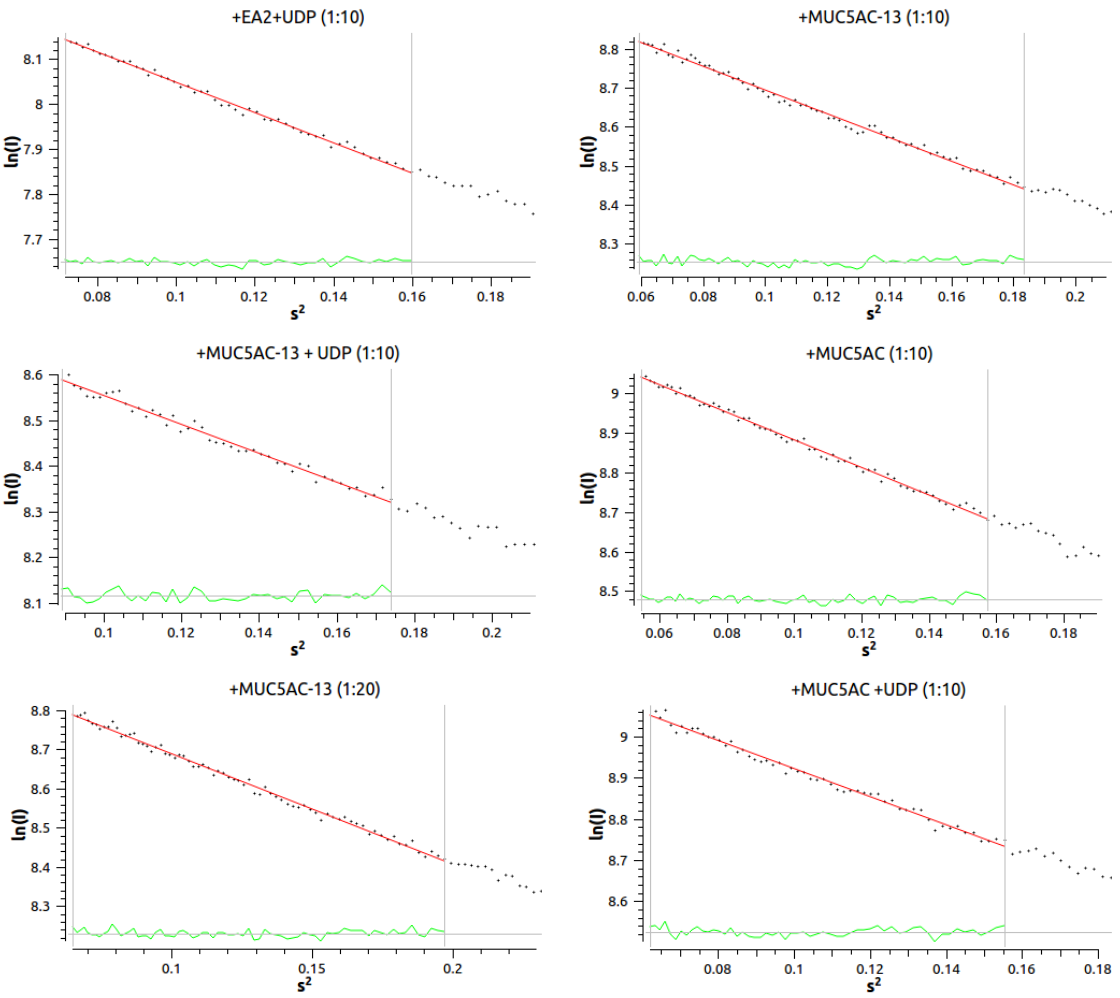

Supplementary Figure 5. Guinier's fits corresponding to samples described in **Supplementary Table 3** . In all the cases, an excellent linear fit is obtained allowing to derive precise radius of gyration, Rg, and forward scattering values, I(0).

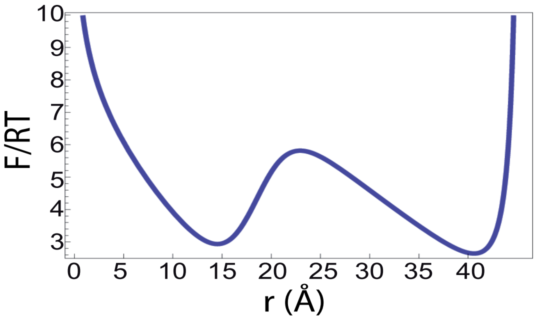

Supplementary Figure 6. Free-energy profile as a function of the end-to-end distance *r* of the flexible linker. The peak at high values of the R_g (R_g corresponding to values of 31-37 Å) is associated to the equilibrium distribution of the WLC model, where a balance between entropy (favoring shorter end-to-end distances *r)* and elastic energy (providing rigidity against bending of the linker) produces a free-energy minimum at high values of *r*. The peak at low values of R_g (R_g corresponding to values of 24.5-29.5 Å) is related to the interaction of the two domains, yielding the minimum at low values of *r*. The values of the parameters used are *lc* = 12 peptide bonds (1 bond = 3.8 A), lp = 0.6 *lc*, r_m = 18.5 Å, r_w =1.8 Å, θ_0 = arccos(0.42), ε/RT=6.4.

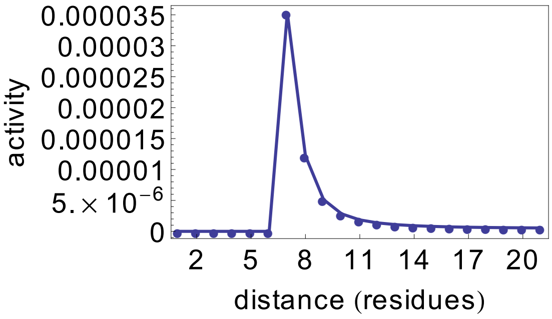

Supplementary Figure 7. Predicted enzymatic activity $\sigma(l,lc)$ (see Supplementary Experimental Procedures) as a function of the residue separation l between the potential acceptor site and a prior fixed glycosite of the glycopeptides. In this plot the flexible linker is fixed to its crystallographic structure. This modification leads to a decrease of ≈ 5,000 fold in activity with respect to that reported in Fig. 5c and changes in the glycosylation profile of the enzyme.

Supplementary Table 1. Name and sequence of the peptides used in this study

Peptide	Sequence
MUC5AC	GTTPSPVPTTSTTSA
MUC5AC-Cys13	GTTPSPVPTTSCT*SA
MUC5AC-13	GTTPSPVPTTSTT*SA
MUC5AC-3-13	GTT*PSPVPTTS TT*SA
MUC5ACs-Cys9	GTTPSPVPC*TS
EA2	DSTTPAPTTK

* indicates a GalNAc moiety linked to the underlined glycosylated amino acid.

Supplementary Table 2. Trytophan fluorescence spectroscopy data.

**Peptides**	**K_ds (μM)**
MUC5AC	7 ± 3
MUC5AC (UDP/Mn^{2+})	31 ± 16
MUC5AC-13	8 ± 3
MUC5AC-13 (UDP/Mn^{2+})	5 ± 1
MUC5AC-3-13	11 ± 4
MUC5AC-3-13 (UDP/Mn^{2+})	17 ± 9

The K_ds were obtained in the absence and presence of UDP (400 μM) and $MnCl_2$ (1 mM) (Supplementary Information).

Supplementary Table 3. Analysis of SAXS data measured for GalNAc-T2 in different experimental conditions.

Ligands (Molar Ratio of GalNAc-T2:Ligands)	GalNAc T2 (mg/ml)	R_g (Å)[e]	R_g (Å)[h]	D_{max} (Å)[f]	MW (kDa)[g]	χ_i^2 mon/dim[i]	χ_i^2 [a]	Ratio of Monomer:Dimer [b]	Ratio of Compact:Extended monomeric structures [c]
	2.5	32.9±0.5	34.8	125	65	1.11/2.03	0.92	61:39	40:60
	5	32.7±0.2	34.2	127	58	1.35/4.49	1.17	72:28	47:53
	10	33.9±0.1	34.4	127	63	3.51/10.19	1.63	62:38	40:60
**UDP (1:5)**	5	32.7±0.3	34.3	121	67	1.81/3.10	0.82	60:40	46:54
**UDP (1:10)**	5[d]	34.4±0.3	35.0	123	68	2.00/4.07	1.00	61:39	42:58
**UDP (1:20)**	5	34.0±0.2	34.5	122	71	2.84/4.66	1.20	56:44	40:60
**UDP (1:50)**	5	36.0±0.3	36.0	129	68	2.73/5.78	1.95	64:36	43:57
**UDP-GalNAc (1:10)**	5[d]	34.7±0.4	34.6	124	62	1.14/3.21	0.76	67:33	40:60
**EA2+UDP (1:10)**	5[d]	31.8±0.2	32.1	112	54	2.15/11.16	1.17	85:15	63:37
**MUC5AC-13 (1:10)**	5[d]	30.2±0.2	30.6	112	50	1.96/7.40	0.74	92:8	70:30
**MUC5AC-13 (1:20)**	5	29.1±0.1	29.2	102	46	2.50/8.88	1.21	100:0	68:32
**MUC5AC-13+UDP (1:10)**	5[d]	30.7±0.4	31.7	112	53	1.60/5.67	1.03	89:11	70:30
**MUC5AC (1:10)**	5[d]	32.3±0.3	33.4	120	59	1.47/5.67	0.79	72:28	59:41
**MUC5AC+UDP (1:10)**	5[d]	32.0±0.2	33.3	120	55	1.39/7.04	0.81	82:18	61:39

(a)- Quality of the EOM fit achieved by using a pool containing monomeric conformations and the PDB entry 2FFV as a dimer. Excellent fits for the SAXS bimodal curve are obtained with low values of χi2 that indicate the pool of GalNAc-T2 conformations represents a good description of the behaviour of the protein in solution.

(b)- Relative percentage of monomers and dimers of GalNAc-T2.

(c)- Relative percentage of compact (R_g < 30.5 Å) and extended (R_g > 30.5 Å) for the monomeric conformations derived from the EOM fit.

(d)- SAXS curves at 1, 2.5 and 10 mg/ml were also measured and analysed. They are omitted here for simplicity.

(e)- Radii of gyration, R_g, determined using Guinier's approach.

(f)- Maximum intramolecular distance, D_{max}, determined from the pair-wise distance distribution, *p(r)*, with the program GNOM.

(g) Estimated molecular weight of the particles in solution based on Porod's volume computed with PRIMUS divided by 1.6. Theoretical MW of GalNAc-T2 is 56.7 kDa.

(h)- Radius of gyration, R_g, derived from the *p(r)* computed with the program GNOM.

(i)- Fitting of the crystallographic monomeric (1FFU) and dimeric (1FFV) structures of GalNAc-T2 to the experimental data using CRYSOL.

Supplementary Methods

Synthesis of peptides and glycopeptides

Our initial rationale to choose the glycopeptides in this work was inspired by an earlier work in which the glycosylation profile of GalNAc-T2 differed depending on whether naked MUC5AC or glycopeptides such as MUC5AC-13, MUC5AC-3 and MUC5AC-3-13 were used as initial substrates (**Supplementary Table 1**). Among other conclusions it was reported that the glycosylation of MUC5AC-13 took place optimally 10 residues N-terminal (Thr3) from the previous glycosylated site Thr13 and suggested to be driven by the lectin domain. Further sites such as Ser5 and Thr9 were also glycosylated but not in an efficient manner[1]. We synthesized 4 glycopeptides to understand the role of the lectin domain in catalysis. While MUC5AC-Cys13 and MUC5AC-13 share the same position of glycosylation (position 13), they differ in the identity of the glycosylated underlying amino acid, a Thr residue in the latter and a Cys residue in the former. An earlier computational study suggested that Cys-S-GalNAc mimicked fairly well the perpendicular conformation of the GalNAc moiety linked to Thr with respect to the backbone peptide[2]. Therefore we introduced a Cys in the peptides to probe experimentally the conformation of the GalNAc moiety in relation to the peptide backbone. The other two glycopeptides, MUC5AC-3-13 and MUC5ACs-C9 (a shorter peptide that also contains a Cys residue linked to a GalNAc moiety), were synthetized to understand how acceptor sites such as Thr3 of MUC5ACs-C9 and Ser5 of MUC5AC-3-13, closely located to previous C-terminal glycosites, are glycosylated.

Computational details

1.1. Initial structure

The crystal structure of GalNAc-T2 in complex with the peptide MUC5AC-Cys13 was used for the computational studies. UDP-GalNAc and the active conformation of the flexible loop, taken from the PDB entry 4D0T, were added to obtain a catalytically productive enzyme complex. To study the glycosylation capabilities of the peptide at different amino acid positions, the peptide was manually extracted from its position in the above complex and placed outside the protein, within a fully solvated environment. Protonation states and hydrogen atom positions of all ionizable amino acids residues were selected base on their hydrogen bond environment. Eight histidine residues were modeled in their neutral states and four in their protonated state. All the crystallographic water molecules were retained and extra water molecules were added to form a 20 Å water box around the protein surface. Nine chloride ions were also added to neutralize the enzyme charge.

1.2 Classical molecular dynamics simulations

MD simulations of the enzyme were performed with the Amber11 software package. The protein was modeled with the FF99SB force field, whereas all carbohydrate molecules were modeled with the GLYCAM06 force field[3]. The MD simulations were carried out in several steps. First, the system was minimized, maintaining the protein, peptide and substrate molecules fixed. In a second step, the entire system was allowed to relax. Weak spatial constraints were initially added to the protein and substrates to gradually reach the desired temperature of 300 K, while the rest of the system was allowed to move freely. The constraints were subsequently removed and the system was subjected to 100 ps of constant pressure MD simulation to adjust the

density of the water environment. Afterwards, 100 ns of constant volume MD simulation were performed. During this time, the peptide molecule remained in a solvated environment without approaching the active site of the enzyme. As no one of the three possible glycosylation positions of the peptide approached the active site during the 100 ns time-scale window, this process was subsequently activated using the metadynamics algorithm[4].

1.2. Classical metadynamics simulations of substrate binding

A snapshot of the equilibrium MD simulation was taken for the metadynamics simulations, which were performed with NAMD2.9 software. As a first approach to model the binding process, only the distance between the center of mass of the side chain of the acceptor amino acid (Thr3, Ser5 and Thr9) and the UDP-GalNAc donor was taken as a collective variable. Therefore three independent metadynamics simulations were carried out for each one of the glycosylation positions. These metadynamics simulations showed a huge variability in the free energy output and did not converge to a stable free energy profile. In an attempt to improve the results, nine additional simulations, with random initial velocities, were launched for each one of the three glycosylation sites. Unfortunately, averaging of the resulting free energy profiles (10 for each glycosylation site) showed large standard deviations. Analysis of the metadynamics trajectories showed that the variability in the results was probably due to the high flexibility of the peptide molecule and the different modes of approaching the active site. To solve this problem, a second collective variable was added to account for the different conformations of the peptide (the RMSD of the C_α carbon atoms of the peptide was chosen). Therefore, three independent metadynamics

simulations were performed with the acceptor amino acid – UDPGalNAc distance and the RMSD of the C_α peptide carbon atoms as collective variables.

The values of the height and width of the Gaussian-like biasing potential were selected as 1.0 kcal·mol^{-1} and 0.25 Å, respectively. A temperature window of ΔT = 25000 K for the well-tempered algorithm was used together with a deposition time of 1 ps. The simulation was continued until the system completely explored the free energy landscape several times which, in terms of the simulation time, corresponds to approximately 100 ns for each metadynamics run.

Atomic Force Microscopy Imaging

In Tapping Mode image operation the cantilever driven by a piezoelectric actuator vibrates near its resonance frequency. Upon approaching the sample, the tip briefly touches the surface at the bottom of each swing, resulting in a decrease in oscillation amplitude. By maintaining constant oscillation amplitude, high-resolution images of the topography of the surface may be obtained on soft samples.

AFM scanning requires the sample be immobilized on a nanoflat surface not to be swept away. Samples of GalNAc-T2 at 2.0 nM in PBS, pH 7.4, were incubated on 1 cm^2 freshly cleaved muscovite mica pieces (Electron Microscopy Sciences) for 10 min at room temperature. The concentration of the protein incubated on the mica sheets was suitable to get isolated features that could be analyzed individually. The enzyme was adsorbed electrostatically on the negatively charged mica surface. GalNAc-T2 presents a net positive charge at the working pH conditions due to its theoretical isoelectric point of 8.3. The immobilization of enzymes on mica was previously evaluated observing clearly they preserve the enzymatic activity[5].

To determine how the ligands affect the conformational dynamics of GalNAc-T2, the enzyme was also incubated with ligands for 10 min under mild stirring at room temperature. UDP, UDP-GalNAc and $MnCl_2$ were used at 20.0 nM and the different peptides were added at 10.0 nM. After sample incubation, the substrate was washed extensively with the same buffer to remove weakly joined molecules. The immobilized sample and the cantilever holder were introduced into a liquid cell (previously cleaned with 20 % isopropanol and Millipore ultrapure water). AFM measurements were conducted also in PBS, pH 7.4, at 20 °C. AFM images were further analyzed by using the WSxM software[6]. Three samples per condition were assayed. At least 10 images of 10 different areas of 500 nm^2 were analyzed for every sample and environment. Furthermore, each feature or associate was analyzed in detail with the zoom function of the WSxM program, performed without losing image information and or the discarding of artifacts.

The height discussed in the main text refers to the Z-height that has sub-nanometric resolution because of the accuracy of piezoelectric scanners. However this does not occur in the X–Y plane, where the scanned features suffer the well documented AFM tip broadening effect that arise in higher sizes[7]. This effect does not affect the comparative analysis of the width related to the size or the conformational state of the protein molecules due to proportionality.

Small-angle X-ray scattering (SAXS) and data analysis

All experiments were performed in a buffer consisting of 25 mM TRIS pH 7.5, 10 mM MnCl2. The first set of experiments contained three different protein concentrations: 10 mg/ml (180 μM), 5 mg/ml (90 μM), 2.5 mg/ml (45 μM) and 1

mg/ml (18 μM). The protein was measured alone and in the presence of ten times molar excess of UDP, UDP-GalNAc, UDP-EA2 peptide, MUC5AC-13, UDP-MUC5AC-13, MUC5AC, and UDP-MUC5AC. The second set of experiments was carried out with the protein concentration fixed at 5 mg/ml, and increasing concentrations of UDP (450 μM, 900 μM, 1.8 mM, 4.5 mM and 9 mM). The concentration of MUC5AC-13 was also changed as follows: 1 mM, 5 mM and 7.5 mM.

Synchrotron SAXS measurements were performed at the European Molecular Biology Laboratory (EMBL) on the storage ring PETRA-III (DESY-Hamburg) on the P12 beamline equipped with a robotic sample changer and a PILATUS-2M. The sample-detector distance was 3.1 m. The scattering intensity, I(s), was recorded at 10ºC in a momentum transfer range of $0.007<s<0.444$ $Å^{-1}$, where $s = (4\pi sin\theta)/\lambda$, 2θ is the scattering angle, and λ = 1.24 Å is the X-ray wavelength. Twenty consecutive 0.05 second measurements were performed for protein samples and buffers to monitor for radiation damage that were subsequently averaged (no systematic radiation effects were observed). Buffer scattering profiles were subtracted from the sample ones using standard procedures [8]. The values of the forward scattering, *I(0)*, and radii of gyration, R_g, were calculated from the experimental SAXS patterns using Guinier's approximation (**Supplementary Fig. 4** and **5**),

$I(s) = I(0)exp(-s^2R_g^2/3)$, which is valid in the range $sR_g < 1.3$.

Structural analysis of the SAXS data was performed using the Ensemble Optimization Method (EOM)[9]. A pool of 10,000 monomeric structures of GalNac-T2 was constructed with the program RanCh[9] by connecting three-dimensional structures of the catalytic and the lectin domains, extracted from the crystallographic structure 2FFU, with a flexible linker of 12 residues with random coil conformations. To

properly describe the data, these monomeric ensembles were enriched with 100 equivalent curves corresponding to one dimeric arrangement. As we tested three dimers, we built three different ensembles with the same 10,000 monomeric conformations and 100 copies of each one of the dimers. The three different dimers used for SAXS are shown in **Supplementary Fig. 3c** and correspond to three different crystal structures obtained under different conditions.

The scattering profiles of all members of the pool were computed with the program CRYSOL using standard parameters[10]. The pool of structures/profiles was submitted to the genetic algorithm (GA) with the aim to select a subensemble of 50 structures/curves with the capacity to collectively describe the experimental SAXS curves. The GA was repeated 200 times starting from different randomly selected subensembles, and the resulting solutions were analysed together. The same pool was used to describe all the measured data. Results were quantitatively analysed by displaying the R_g distributions of the selected ensembles and the relative population of monomeric and dimeric species.

Three different dimeric arrangements were introduced individually in the pool. In all three cases the dimers display similar contacts between the lectin domains and the lectin domain of one monomer with the catalytic domain of a second monomer (**Supplementary Fig. 3c**). We can distinguish the crystallographic dimers by the buried area parameter inferred from the PISA server. The dimers present in the crystal structure of GalNAc-T2-UDP complex (PDB entry 2FFV) are the most compact ones with a buried area of 1689 Å^2 followed by dimers from the PDB entry 4D0T with 1400 Å^2 of buried area, and finally dimers from the crystal structures with the tetragonal space group that in turn appear to be the most flexible compact structures of all dimers with ≈ 750 Å^2 of buried area. Taking into account these dimers for the

SAXS analysis, it was clear that the most rigid dimeric form belonging to the PDB entry 2FFV fitted better to the data (c2 = 1.3 versus values > of 1.5 for other dimeric structures).

Theoretical modeling of the SAXS results

To reproduce the SAXS distribution of the radius of gyration, we consider the crystal structure of GalNAc-T2-UDP-MUC5AC-13 complex, and define the linker corresponding to residues 437-449 (where A and B correspond to the alpha-carbon atoms of residues 437 and 449, respectively; **Fig. 5a**).

To generate the model, we made the following assumptions:

1) The catalytic (residues 75-436) and lectin domains (449-569) are rigid bodies. For the sake of simplicity and to avoid introducing three more variables corresponding to the Euler angles of rotation, their relative orientation is fixed to that found in the crystal structure of GalNAc-T2-UDP-MUC5AC-13 complex.

2) The linker residues are "phantoms", meaning that neither contribute to the radius of gyration, nor interact with each other or with the rest of the protein.

3) The linker is described as a Worm-Like-Chain (WLC)[11], which is a classical model of polymer physics for semi-flexible polymers. In WLC a polymer is represented as a continuous curve, with just an elastic energy, opposed to bending. At any temperature, its equilibrium configurations are the result of a trade-off between the elastic energy that favours stretched and loopy conformations, and entropy. Thus the

behavior of the WLC can be characterized by specifying just the contour length $l_c$and the persistence length l_p. The latter is a measure of the rigidity of the curve: roughly, two portions of the chains which are more than $2l_p$away can have any relative orientation, while below that separation, their orientations will be correlated.

4) According to the coarse-grained spirit of the model, we describe the interaction between the two domains by simply adding a sigmoidal potential $E(r)$ that depends on the distance between the ends of the linker $r = |r| = |B - A|$(**Fig. 5a**):

$$E(r) = -\epsilon\left(1 + exp\left[\frac{1}{r_w}(r - r_m)\right]\right)^{-1}.$$

Such potential is $-\epsilon$at r=0; it halves itself at $r = r_m$and then goes to 0 for increasing r; the smaller r_w, the sharper the transition. Here, r_m and r_w are regarded as adjustable parameters. We verified that the results, at the qualitative level, do not depend strongly on the choice of the potential.

5) The WLC is an isotropic model: setting an end of the polymer at the origin of the reference frame, there is no intrinsically angular preference for the position of the other end, that will be spherically distributed around the origin. We limit the angular freedom in our model, by restricting the second end of the linker within a cone "in front of" the catalytic domain (in a sense specified below), thus preventing configurations where the lectin domain is situated opposite to the catalytic one (**Fig. 5a**).

In this framework, the radius of gyration $R_g(X)$ of any protein conformation X will become a function of the distance vector r. Indeed, denoting $r_i as$ the position of atom

"i" of the protein in conformation X, we can write the following equation that neglects the contributions from the linker residues:

$$R_g^2(X) = \frac{1}{2N^2}\sum_{i,j=k_1}^{k_L}\left(r_j - r_i\right)^2 = \frac{N_A^2}{N^2}R_{g,A}^2 + \frac{N_B^2}{N^2}R_{g,B}^2 + \frac{1}{N^2}\sum_{i=k_1}^{k_A-1}\sum_{j=k_B+1}^{k_L}\left(r_j - r_i\right)^{2,}$$

where k_1,k_A,k_B,$k_L$are the indices of the first atom of the protein, first atom of the linker, last atom of the linker, and last atom of the protein, respectively, and

$$R_{g,A}^2 = \frac{1}{2N_A^2}\sum_{i,j=k_1}^{k_A-1}\left(r_j - r_i\right)^2,$$

$$R_{g,B}^2 = \frac{1}{2N_B^2}\sum_{i,j=k_B+1}^{k_L}\left(r_j - r_i\right)^2,$$

are the radii of gyration of just the catalytic (A) domain and lectin (B) domain, respectively; here N_A , N_B are the number of atoms in domain A and B, and $N = N_A + N_B$.

Upon introducing the coordinates of the centers of the two domains as $C_1 = \frac{1}{N_A}\sum_{i=k_1}^{k_A-1} r_i$and $C_2 = \frac{1}{N_B}\sum_{i=k_B+1}^{k_L} r_i$, and the distances of the ends of the linker from the centers: $x_A = A - C_1$,$x_B = B - C_2$, we can write:

$$r_i = \begin{cases} A + d_i = C_1 + x_A + d_i \; if \; i = k_{1,} \dots, k_A - 1 \\ B + \delta_i = C_2 + x_B + \delta_i \; if \; i = k_B + 1, \dots, k_L \end{cases}$$

Substituting the above equations in the expression for the radius of gyration, we can relate the latter to the end-to-end vector of the linker, $r = B - A$, as

$$R_g^2(r) = \Pi + \frac{N_A N_B}{N^2}(r^2 + 2rR),$$

where $R = x_A - x_B$and

$$\Pi = \frac{1}{N^2}\left(N_A^2 R_{g,A}^2 + N_B^2 R_{g,B}^2 + N_A N_B \sum_{i=k_1}^{k_A-1} \sum_{j=k_B+1}^{k_L} \left(\delta_j - d_i\right)^2 \right),$$

is independent from r. The above expression for the radius of gyration allows to relate the probability distribution for the latter, as revealed by SAXS, to the probability density of the end-to-end vector of the linker. The latter will be given by

$$f(r|lc, lp) = \frac{1}{Z(lc, lp)} Q^{WLC}\left(\frac{r}{lc}, \frac{lp}{lc}\right) exp\left(-\frac{E(r)}{RT}\right) Y(\vartheta, \phi)$$

where $Q^{WLC}\left(\frac{r}{lc}, \frac{lp}{lc}\right)$is the distribution for the end-to-end distance in WLC as reported in Eq. 21 by Becker *et al*[12]. The exponential represents the Boltzmann factor due to the interaction and $Y(\vartheta, \phi)$ is the angular probability distribution, that we add to the isotropic WLC as discussed in item 5 above.

In the spirit of the coarse-grained model, and for the sake of simplifying the calculations, we use spherical coordinates, selecting R as the polar direction for the angle ϑ (we have verified that the angle between R and the $C_2 - C_1$axis is reasonably small, around 18 degrees), and keep isotropy for rotations of an angle ϕaround the polar axis, so that

$$Y(\vartheta, \phi) = \begin{cases} 1 \; if \vartheta < \vartheta_0 \\ 0 \; if \vartheta \geq \vartheta_0 \end{cases}$$

Finally, $Z(lc, lp) = \int r^2 \, sin(\vartheta) dr d\vartheta d\phi f(r|lc, lp)$ is the partition function, that ensure the proper normalization of the probability.

The probability distribution of the radius of gyration can be derived from $f(r|l, lc)$by performing a change of variables

$r = (r, \vartheta, \phi) \rightarrow y = \left(y = R_g(r, \vartheta), \vartheta' = \vartheta, \phi' = \phi\right)$

and imposing that the density-function in the new variables describe the same probability of finding the end-to-end vector within a certain volume element dV:

$$g(y|lc, lp)dV'(y, \vartheta', \phi') = f(r|lc, lp)dV(r, \vartheta, \phi),$$

whence

$$g(y|lc, lp) \equiv g(y, \vartheta'|lc, lp) = f(r(y, \vartheta), \vartheta|lc, lp)r^2(y, \vartheta)\left|\frac{\partial(r, \vartheta, \phi)}{\partial(y, \vartheta', \phi')}\right|,$$

with $\left|\frac{\partial(r,\vartheta,\phi)}{\partial(y,\vartheta',\phi')}\right|$the Jacobian determinant of the change of variables. Finally we get:

$$g(y, \vartheta'|l, lc) = f(r(y, \vartheta), \vartheta|l, lc)\frac{N^2}{N_A N_B y}\frac{r^2(y, \vartheta)}{\left(r(y, \vartheta) + Rcos(\vartheta)\right)},$$

The population of the radius of gyration observed in the experiments will be given by:

$$G(R_g) = 2\pi R_g^2 \int sen\,(\vartheta')d\vartheta' g\left(y = R_g, \vartheta'|lc, lp\right).$$

Modeling the enzymatic activity

To the hypotheses above, we add another assumption:

6) To consider the enzymatic activity, we introduce a ligand peptide bound at P_2, again described as a WLC.

To this end, we consider points P_1, P_2 (**Fig. 5a**) that are located in the active site and the α subdomain of the lectin domain, respectively, and correspond to the position of the alpha carbons of the acceptor and prior glycosylated sites.

We then let the protein domains and ligand, move freely, with the only constraint that the ligand is always bound to the lectin domain at P_2. The glycosylation reaction at P_1 will take place when the linker and ligand conformation are such that the acceptor site of the ligand is found at its "active site" position P_1 in the catalytic domain.

The rate at which the reaction takes place will depend on the dynamics of the ligand and protein, as well as on the distance (in residues) between the prior glycosylated and the acceptor sites of the ligand.

We thus estimate the enzymatic activity as a function of the prior glycosylated and the acceptor sites distance that relies on the product of the equilibrium probability for the linker and the ligand.

Upon introducing, as above, $d_{P_1} = P_1 - A$,$d_{P_2} = P_2 - B$, and remembering that the linker end-to-end distance is $r = B - A$, we start by defining:

$$\rho_G(r) = P_1 - P_2 = d_{P_1} - \delta_{P_2} - r$$

which is the "correct" distance vector between the prior glycosylated and the acceptor sites on the ligand for the reaction to take place; such vector depends on the protein conformation through the linker end-to-end vector r. For a ligand peptide with the the prior glycosylated and the acceptor sites separated by l residues, the probability that the glycosylation residues are found in a volume dV_ρ around a distance vector ρ, when the protein linker end-to-end distance vector is found in volume dV_r around r, is:

$$dp = \xi(r, \rho | l, lc) dV_\rho dV_r,$$

For the sake of simplicity, we will assume that the density $\xi(r, \rho | l, lc)$ is simply the product of the separate distributions for the linker and ligand, as if they were

statistically independent, and the conformation of the former wouldn't affect the variability of the latter. Moreover, we ignore any angular bias on ρ, as if the ligand's free end were completely free to move in any direction, and assume a WLC behavior for the ligand, too.

This yields $\xi(r,\rho|l,lc) = f^{WLC}(\rho|l,\lambda)f(r|lc,lp)$, where the latter factor is the equilibrium probability for the linker, and the former is the isotropic WLC expression for the ligand, assuming a persistence length λ for the latter:

$$f^{WLC}(\rho|l,\lambda) = \frac{Q^{WLC}(\rho/l,\lambda/l)}{4\pi \int d\rho\rho^2 Q^{WLC}(\rho/l,\lambda/l)}.$$

Finally, the probability that, for a given length lc of the linker and a separation l between the prior glycosylated and the acceptor sites in the ligand, the latter meet the correct sites on the protein, for any protein conformation, is found by imposing $\rho = \rho_G(r)$ and integrating over the linker conformations:

$$\sigma(l,lc) = \int d^3 r \xi(r,\rho = \rho_G(r)|l,lc)$$

To dissect the role of the linker and the ligand flexibility in determining the activity, we consider the extreme case of the linker completely frozen in its crystallographic conformation. In this case, the above equation for $\sigma(l,\lambda)$ is replaced by:

$$\sigma(l) = f^{WLC}(\rho = \rho_G(r_0)|l,\lambda)$$

and depends on the separation "l"of the prior glycosylated and the acceptor sites of the peptide (in the above equation $r_0$is the end-to-end distance of the linker in the crystal structure).

Supplementary References

1 Raman, J. *et al.* The catalytic and lectin domains of UDP-GalNAc:polypeptide alpha-N-Acetylgalactosaminyltransferase function in concert to direct glycosylation site selection. *J Biol Chem* **283**, 22942-22951, doi:10.1074/jbc.M803387200 (2008).

2 Aydillo, C. *et al.* S-Michael additions to chiral dehydroalanines as an entry to glycosylated cysteines and a sulfa-Tn antigen mimic. *J Am Chem Soc* **136**, 789-800, doi:10.1021/ja411522f (2014).

3 Kirschner, K. N. *et al.* GLYCAM06: a generalizable biomolecular force field. Carbohydrates. *J Comput Chem* **29**, 622-655, doi:10.1002/jcc.20820 (2008).

4 Laio, A. & Parrinello, M. Escaping free-energy minima. *Proc Natl Acad Sci U S A* **99**, 12562-12566, doi:10.1073/pnas.202427399 (2002).

5 Marcuello, C., de Miguel, R., Gomez-Moreno, C., Martinez-Julvez, M. & Lostao, A. An efficient method for enzyme immobilization evidenced by atomic force microscopy. *Protein Eng Des Sel* **25**, 715-723, doi:10.1093/protein/gzs086 (2012).

6 Horcas, I. *et al.* WSXM: a software for scanning probe microscopy and a tool for nanotechnology. *Rev Sci Instrum* **78**, 013705, doi:10.1063/1.2432410 (2007).

7 Meinander, K., Jensen, T. N., Simonsen, S. B., Helveg, S. & Lauritsen, J. V. Quantification of tip-broadening in non-contact atomic force microscopy with carbon nanotube tips. *Nanotechnology* **23**, 405705, doi:10.1088/0957-4484/23/40/405705 (2012).

8 M. V. Petoukhov, D. F., A. V. Shkumatov, G. Tria, A. G. Kikhney, M. Gajda, C. Gorba, H. D. T. Mertens, P. V. Konarev and D. I. Svergun. New developments in the ATSAS program package for small-angle scattering data analysis. *J. Appl. Cryst.* **45**, 9 (2012).

9 Bernado, P., Mylonas, E., Petoukhov, M. V., Blackledge, M. & Svergun, D. I. Structural characterization of flexible proteins using small-angle X-ray scattering. *J Am Chem Soc* **129**, 5656-5664, doi:10.1021/ja069124n (2007).

10 Svergun D.I., B. C. a. K. M. H. J. CRYSOL - a Program to Evaluate X-ray Solution Scattering of Biological Macromolecules from Atomic Coordinates *J. Appl. Cryst.* **28**, 6 (1995).

11 O. Kratky, G. P. Röntgenuntersuchung gelöster Fadenmoleküle. *Rec. Trav. Chim.* **68**, 18 (1949).

12 Becker, N. B., Rosa, A. & Everaers, R. The radial distribution function of worm-like chains. *Eur Phys J E Soft Matter* **32**, 53-69, doi:10.1140/epje/i2010-10596-0 (2010).
